# The chloroplast genome of *Chrozophora sabulosa* Kar. & Kir. and its exploration in the evolutionary position uncertainty of genus *Chrozophora*

**DOI:** 10.1186/s12864-024-10366-3

**Published:** 2024-06-14

**Authors:** Nida Javaid, Musarrat Ramzan, Shagufta Jabeen, Yanjun Du, Muhammad Anwar, Song Xiqiang

**Affiliations:** 1 School of Tropical Agriculture and Forestry (School of Agriculture and Rural Affairs,School of Rural Revitalization), Hainan University, Haikou, P.R. China; 2 Key Laboratory of Genetic and Germplasm Innovation of Tropical Special Forest Trees and Ornamental Plants, Ministry of Education, Hainan University, Haikou, P.R. China; 3 The Islamia University, Bahawalpur, Pakistan; 4 Haikou, P.R. China

**Keywords:** Euphorbiaceae, Phyllanthaceae, *Chrozophora sabulosa*, Chrozophoreae, Phylogenetic findings

## Abstract

*Chrozophora sabulosa* Kar. & Kir. is a biennial herbaceous plant that belongs to the Euphorbiaceae family and has medicinal properties. This research aimed to identify the genetic characteristics and phylogenetic position of the *Chrozophora* genus within the Euphorbiaceae family. The evolutionary position of the *Chrozophora* genus was previously unknown due to insufficient research. Therefore, to determine the evolutionary link between *C. sabulosa* and other related species, we conducted a study using the NGS Illumina platform to sequence the *C. sabulosa* chloroplast (cp.) genome. The study results showed that the genome was 156,488 bp in length. It had a quadripartite structure consisting of two inverted repeats (IRb and IRa) of 24,649-bp, separated by an 87,696-bp LSC region and a 19,494-bp SSC region. The CP genome contained 113 unique genes, including four rRNA genes, 30 tRNA genes, and 79 CDS genes. In the second copy of the inverted repeat, there were 18 duplicated genes. The *C. sabulosa* lacks the *petD*, *petB*, *rpl2*, and *rps16* intron. The analysis of simple sequence repeats (SSRs) revealed 93 SSR loci of 22 types and 78 oligonucleotide repeats of four kinds. The phylogenetic investigation showed that the *Chrozophora* genus evolved paraphyletically from other members of the Euphorbiaceae family. To support the phylogenetic findings, we selected species from the Euphorbiaceae and Phyllanthaceae families to compare with *C. sabulosa* for Ks and Ka substitution rates, InDels investigation, IR contraction and expansion, and SNPs analysis. The results of these comparative studies align with the phylogenetic findings. We identified six highly polymorphic regions shared by both families, which could be used as molecular identifiers for the *Chrozophora* genus (*rpl33-rps18*, *rps18-rpl20, rps15-ycf1*, *ndhG-ndhI*, *psaI-ycf4*, *petA-psbJ*). The cp. genome sequence of *C. sabulosa* reveals the evolution of plastid sequences in *Chrozophora* species. This is the first time the cp. genome of a *Chrozophora* genus has been sequenced, serving as a foundation for future sequencing of other species within the Chrozophoreae tribe and facilitating in-depth taxonomic research. The results of this research will also aid in identifying new *Chrozophora* species.

**Supplementary Information:**

The online version contains supplementary material available at 10.1186/s12864-024-10366-3.

## Introduction

The Euphorbiaceae family has approximately 9,000 species spread across 340 genera and 52 tribes, primarily found in various tropical regions [1–7]. The genus *Chrozophora* A. Juss. belongs to the Chrozophoreae tribe of the Acalyphoideae subfamily of the Euphorbiaceae family [8]. *Chrozophora* is found in the Mediterranean, tropical Africa, and West Asia, as well as in Pakistan’s tropical and temperate zones [2]. According to “World Flora Online” http://www.worldfloraonline.org/taxon/wfo-4000008162: *Chozophora* has 9 accepted species (*C. gangetica* Gand., *C. brocchiana* Schweinf., *C. mujunkumi* T. Nasimova, *C. oblongifolia* (Delile) A. Juss. ex Spreng., *C. plicata* (Vahl) A. Juss. ex Spreng., *C. rottleri* (Geiseler) Spreng., *C. sabulosa* Kar. & Kir., *C. senegalensis* (Lam.) Spreng., *C. tinctoria* (L.) A. Juss.). *Chrozophora sabulosa* Kar & Kir, known as Nilakari, is an important medicinal plant from the Euphorbiaceae family [1].

The family Euphorbiaceae is challenging to understand due to its wide distribution range, many species, and poorly defined genera. However, it has been confirmed that Euphorbiaceae is monophyletic based on molecular and embryological features [8–10]. Webster [6] has classified the Euphorbiaceae family into five subfamilies based on the number of ovules per ovary locule: Phyllanthoideae, Oldfieldioideae, Acalyphoideae, Crotonoideae, and Euphorbioideae. In the Angiosperm Phylogeny Group (APG II 2003) [11] classification, the family was divided into four groups: Euphorbiaceae s.s., Phyllanthaceae, Picrodendraceae, and Putranjivaceae, all in the clade Malpighiales. The subfamilies with uniovulate ovary locules (Euphorbioideae, Acalyphoideae, and Crotonoideae) are considered Euphorbiaceae s.s [11]. The family was further divided into several subfamilies and tribes based on molecular data [12], and some genera were moved to independent families [5]. APG III (2009) [13] divides it into four subfamilies: Acalyphoideae, Cheilosoideae, Crotonoideae, and Euphorbioideae. Despite the extensive research carried out by botanists, who have conducted studies in taxonomy, anatomy, phytochemistry, economic botany, and molecular systematic, the knowledge of this family still has significant gaps, even regarding morphology [5]. Detailed molecular, morphological, and anatomical studies involving many genera are required to propose a safer classification for this family. However, some genera within the family, such as *Chrozophora*, still have confusing taxonomic positions. To understand the evolutionary relationship between *C. sabulosa* and related plants, it is necessary to sequence the chloroplast genomes from the *Chrozophora* genus and the Chrozophoreae tribe.

Previous studies have used both molecular and morphological data to perform phylogenetic reconstructions. However, it has been suggested that molecular approaches are more reliable in phylogenetics [8–10]. Among molecular approaches, cp. genome sequences have gained significant interest in plant phylogenetics, phylogeography, and molecular evolution investigations in recent years [14]. The cp. genome has a significantly conserved gene content and genome order [15]. Moreover, it is smaller, has fewer nucleotide alterations, and has fewer genome sequence reorganizations than the nuclear and mitochondrial genomes. These characteristics make it an excellent tool for understanding genome evolution in complicated angiosperm families [14–18]. As a result, cp. genomes provide valuable data that can be easily combined with source molecular data to validate complicated evolutionary connections and perform comprehensive phylogenetic analyses [17]. More than 400 species of Euphorbiaceae have had their cp. genomes sequenced using a high-throughput sequencing technique [10]. However, data on the chloroplast genomes of *Chrozophora* was unavailable, which limits further information regarding its phylogenetic position in the family. Additionally, no members of the Chrozophoreae tribe have had their cp. genomes sequenced yet, which casts doubt on the tribe’s exact phylogenetic position. The present study aimed to investigate the cp. genome of *C. sabulosa* to clarify the evolutionary position of the *Chrozophora* genus in the Euphorbiaceae family. By contributing significant molecular and phylogenetic data on the *Chrozophora* genus, this study may aid in species identification and determination of its evolutionary position. The *C. sabulosa* cp. genome has been marked as the first member of the *Chrozophora* genus and the Chrozophoreae tribe to be sequenced. The results of this research may provide a foundation for phylogenetic investigations of the Chrozophoreae tribe.

## Results

### *C. sabulosa* cp Genome assembly and its characteristics

**Fig. 1:**
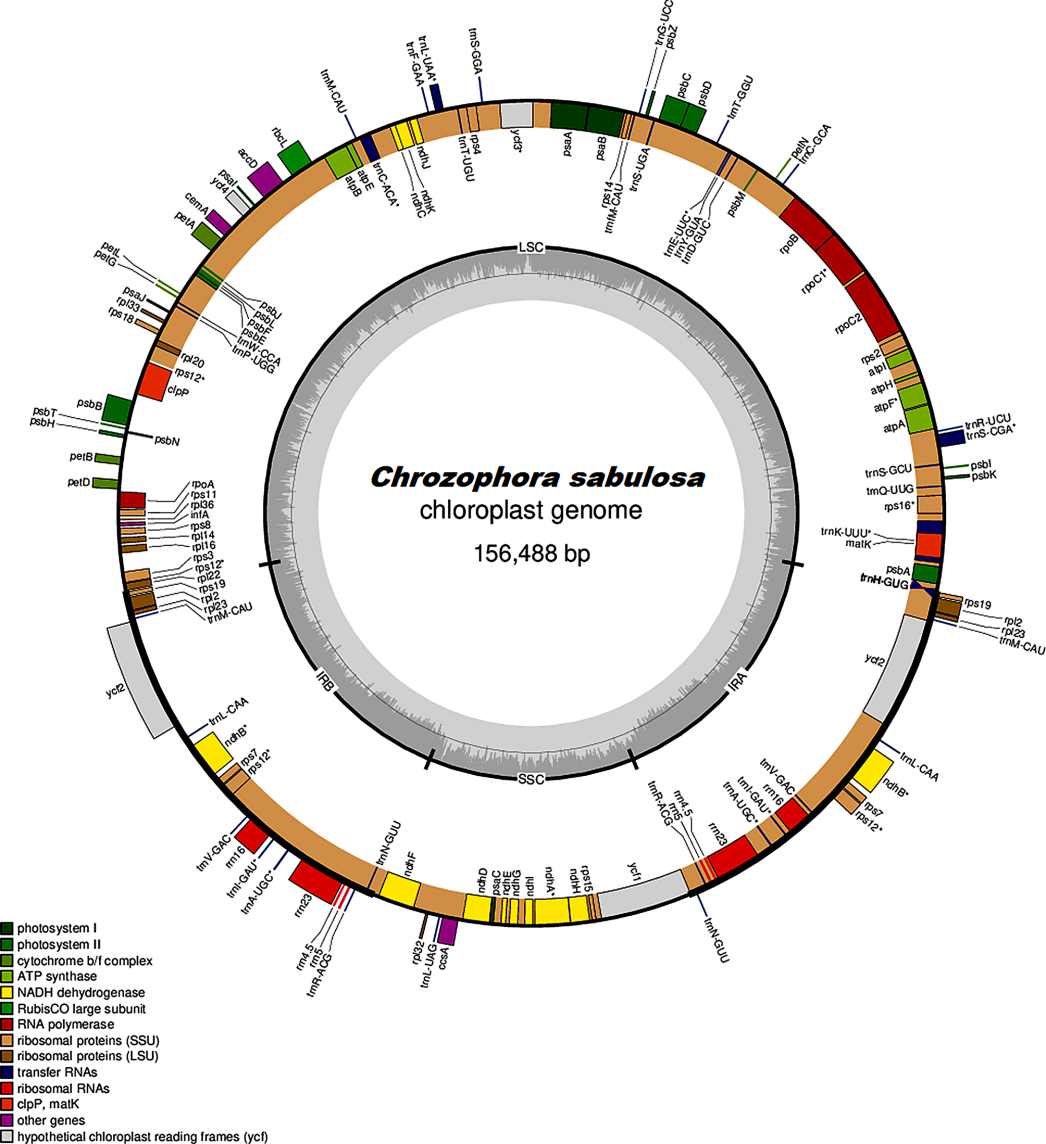
The cp. genome map of *C. sabulosa*. Genes outwards the map is demonstrated clockwise, while inside genes are transcribed anticlockwise. Color coding is used to differentiate between functional groups of genes. The intensity of the inner circle color indicates the amount of GC (Dark grey) and AT (Light grey). LSC denotes the large single copy, SSC denotes the small single copy, and IRb and IRa denote inverted repeats

The Illumina HiSeq2500 generated 10.1 GB of raw data for *C. sabulosa* through paired-end sequencing with 150 bp reads. The de novo assembled cp. genome had an average coverage depth of 271 and was 156,488 bp long, comprising two inverted repeats (IRb and IRa) of 24,649 bp, an LSC region of 87,696 bp, and an SSC region of 19,494 bp (Fig. 1). The total GC content of *C. sabulosa* was 36.5%, with the highest GC content (43.3%) found in the IRs, followed by 34.1% in the LSC region and 30% in the SSC region. The CP genome contained 113 distinct genes, including 79 CDS genes, four rRNA genes, and 30 tRNA genes, as shown in Table 1. The LSC segment had 84 genes, while the SSC segment had 13. Table S1 listed the 15 genes (out of 113) with introns, including three with two introns (*clpP*, *ycf3*, *rps12*), five tRNA, and five CDS genes with one intron. The *rps12* gene was repeated twice, leading to a trans-splitting event. The *psbL* gene in *C. sabulosa* began with a TCG codon, resulting in Threonine as the first amino acid. The cp. genome lacked the *petB*, *petD*, *rpl16*, and *rpl2* introns. Table 2 provides a detailed description of the genes in *C. sabulosa* based on their function.

**Table 1:** The cp. genome of *Chrozophora sabulosa* is described in detail

Category	Items	Descriptions
**Construction of cp. genome**	Length of LSC	87,696 bp
	Length of SSC	19,494 bp
	Length of IRs (IRA, IRB)	24,649 bp
	Complete Genome Size	156,488 bp
**Gene content**	Total number of genes	131
	No. of Protein-coding genes	86
	No. of tRNAs	37
	No. of rRNAs	8
	No. of genes in LSC	84
	No. of genes in SSC	13
	No. of genes with two copies in IRs	18
	Total genes length	104,727 bp
	The average length of genes	799 bp
	Gene length/Genome ratio	0.67%
**GC content percentage**	GC % in LSC	34.10%
	GC % in SSC	30.00%
	GC % in IR	43.30%
	GC content (%) overall	36.50%
**Intron containing genes**	Total intron-containing genes	15
	ICGs Protein coding (CDS)	10
	ICGs in tRNA	5
	ICGs in rRNA	0
	1 Intron containing Genes	11
	2 Intron-containing Genes	*clpP*, *ycf3*, *rps12*, *rps12*

**Table 2:** *Chrozophora sabulosa* gene functions are summarized in this table

The primary category of genes	Functional group of genes
Genes involved in photosynthesis	Photosystem subunits (*ndhI*, *ndhJ*, *ndhK*, *psaA*, *psaB*, *psaC*, *psaI*, *psaJ*, *psbA*, *psbB*, *psbC*, *psbD*, *psbE*, *psbF*, *psbH*, *psbI*, *psbJ*, *psbK*, *psbL*, *psbM*, *psbN*, *psbT*, *psbZ*)
	Cytochrome b/f complex subunits (*petA*, *petB*, *petD*, *petG*, *petL*, *petN*)
	Hypothetical chloroplast RF1 (*ycf1*)
	Photosystem I assembly protein Ycf3 (*ycf3*)
	Photosystem I assembly protein Ycf4 (*ycf4*)
	Rubisco large subunit (*rbcL*)
	ATP synthase subunits (*atpA*, *atpB*, *atpE*, *atpF*, *atpH*, *atpI*)
	NADH dehydrogenase subunits (*ndhA*, *ndhB*, *ndhC*, *ndhD*, *ndhE*, *ndhF*, *ndhG*, *ndhH*)
Self-replicating genes	rRNA genes (*rrn16*, *rrn23*, *rrn4.5*, *rrn5*)
	tRNA genes (*trnH-GUG*, *trnK-UUU*, *trnS-CGA*, *trnC-ACA*, *trnL-UAA*, *trnA-UGC*, *trnS-UGA*, *trnI-GAU*, *trnM-CAU, trnS-GCU*, *trnS-GGA*, *trnY-GUA*, *trnW-CCA*, *trnL-CAA*, *trnL-UAG*, *trnP-UGG*, *trnD-GUC*, *trnfM-CAU*, *trnR-ACG*, *trnT-UGU*, *trnM-CAU*, *trnE-UUC*, *trnF-GAA*, *trnC-GCA*, *trnT-GGU*, *trnQ-UUG*, *trnR-UCU*, *trnV-GAC*, *trnN-GUU*, *trnG-UCC*)
	Ribosome small subunit (*rps11*, *rps12*, *rps14*, *rps15*, *rps16*, *rps18*, *rps19*, *rps2*)
	Ribosome large subunit (*rps3*, *rps4*, *rps7*, *rps8*, *rpl2*, *rpl16*, *rpl20*, *rpl22*, *rpl14*, *rpl23*, *rpl32*, *rpl33*)
	DNA-dependent RNA polymerase (*rpoA*, *rpoB*, *rpoC1*, *rpoC2*, *rpl36*)
Other genes	Maturase (*matK*)
	Envelope membrane protein (*cemA*)
	acetyl-CoA subunit (*accD*)
	C-type cytochrome synthesis gene (*ccsA*)
	Translational initiation factor (*infA*)
	Protease (*clpP*)
	Conserved open reading frames (*ycf2*)

### RSCU, and amino acid frequencies in *C. sabulosa*

**Fig. 2:**
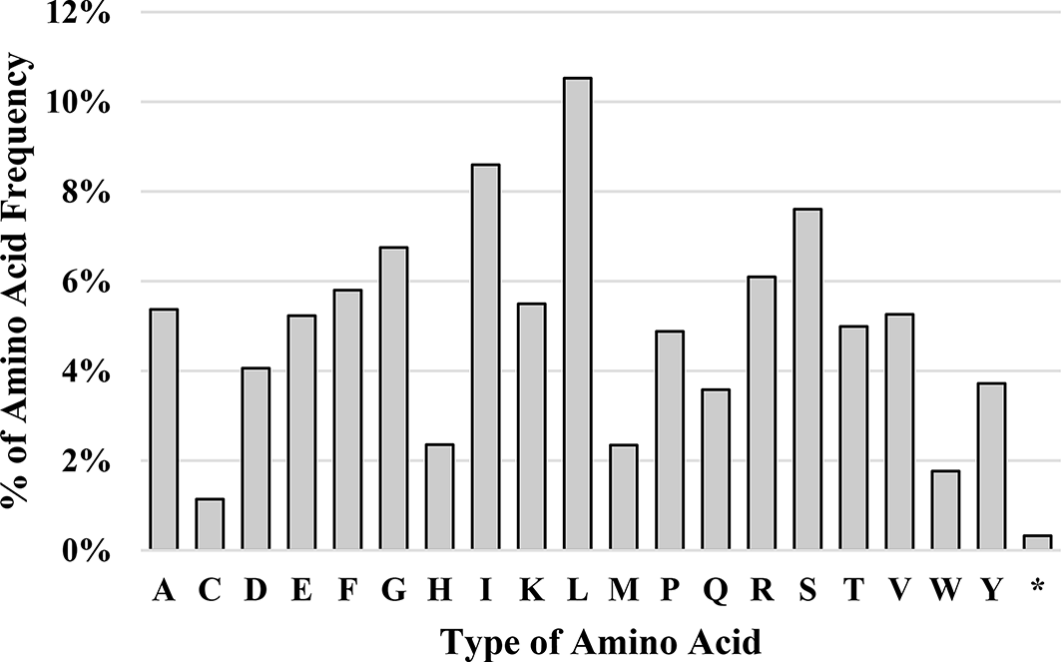
Amino acids frequency (%) of *C. sabulosa*

All genomes showcase codon usage bias, which affects translational dynamics, consistency, accuracy, and protein folding [19]. The Relative synonymous codon usage (RSCU) ratio is the average usage frequency of a codon divided by its predicted unbiased usage frequency. Recent studies have highlighted the significance of codon usage in the evolution of the cp. genome [20, 21]. In the coding sequences in *C. sabulosa*, there are 52,162 codons within 79,461 bp. The most abundant amino acid in the cp. genome of *C. sabulosa* is leucine (11%), followed by isoleucine (9%), and the least abundant is cysteine (1%) (for further details, see Table S2 and Fig. 2). We have identified 31 variant codons with RSCU values greater than one, indicating that *C. sabulosa* uses them specifically to encode certain amino acids. The AGA codon, which codes for arginine, has the highest usage bias (2.05), while the CGC codon, which also codes for arginine, has the lowest (0.44). There was no bias at codons AUG (Methionine), CCC (Proline), and UGG (Tryptophan) in *C. sabulosa* cp. genomes with 1.00 RSCU (Table S3).

### Editing sites of RNA

**Fig. 3:**
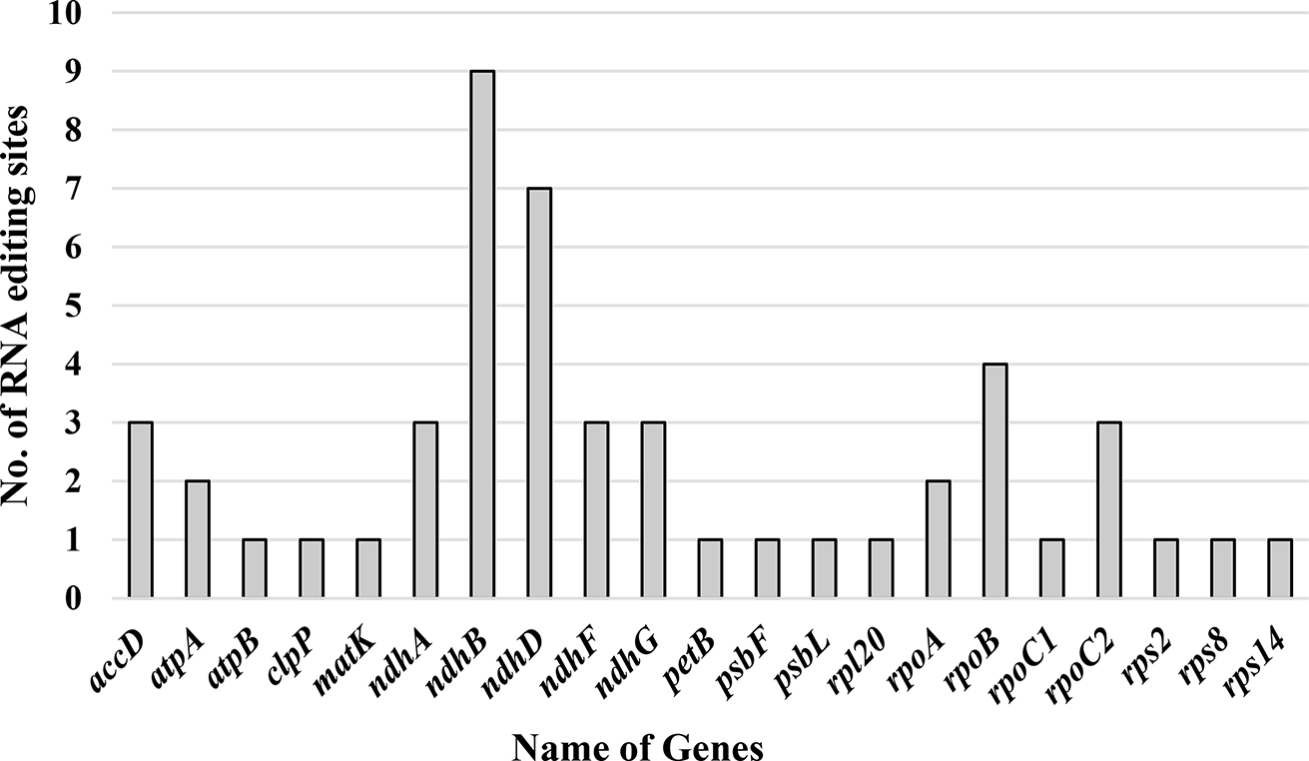
RNA editing sites of *C. sabulosa* cp. genome

PREP-cp detected 50 RNA editing sites in 21 genes from *C. sabulosa*. The *ndhB* gene has the highest number of RNA editing sites (9 sites), followed by the *ndhD* gene (7 sites) (refer to Fig. 3). Modifying a nucleotide in the first position of a codon resulted in 13 editing sites (26%), while changes in the second position resulted in 37 editing sites (74%). Most RNA editing sites (42%) were found in Serine codons. In *C. sabulosa*, 90% of the Serine was converted to Leucine, while the remaining 10% was converted to Phenylalanine. The codon-encoded Proline had the second-highest conversion rate (18%), while the codon-encoded Threonine had the third-highest conversion rate (14%). Proline, Serine, Threonine, and Alanine showed multiple types of nucleotide conversion, whereas Leucine, Arginine, and Histidine showed only one. Hydrophobic amino acid conversions [including Proline (9), Alanine (2), and Leucine (6)] occurred at 34% of all RNA editing sites. In contrast, soluble amino acids resulted in 33 conversions (66%), including Histidine (3), Threonine (7), Serine (21), and Arginine (2). Three non-polar to polar conversions, 14 non-polar to non-polar conversions, 30 polar to non-polar conversions, and three polar-to-polar amino acid conversions have also been found. For further information, please refer to Table S4, which details all the RNA editing sites.

### Detecting simple sequence repeats (SSRs) in *C. sabulosa*

**Fig. 4:**
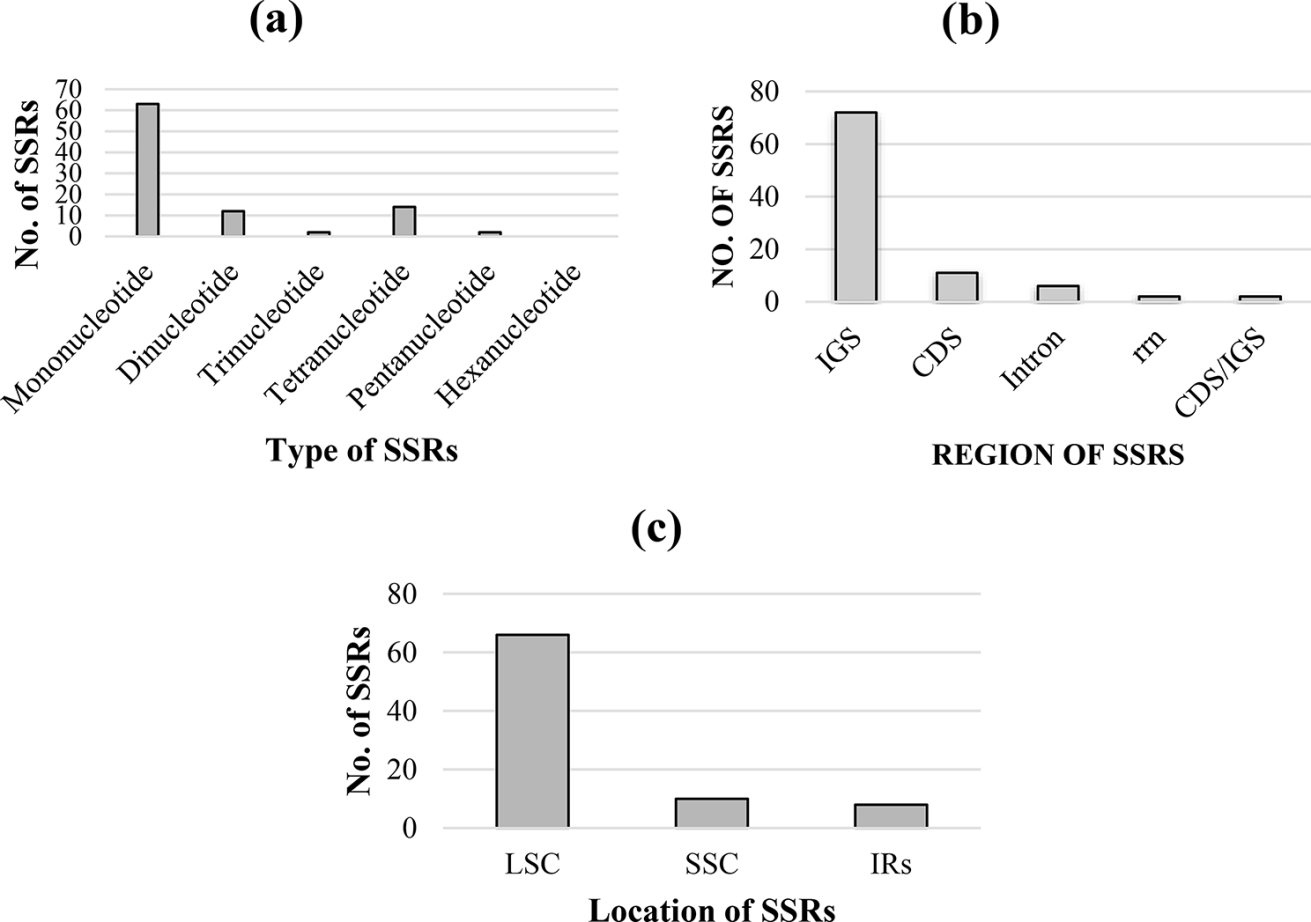
SSR analysis of C. sabulosa. (**a**) Types of SSRs. (**b**) Distribution of SSRs in active cp. genome regions. (**c**) Location of SSRs

In this study, MISA obtained a set of 93 SSRs, which had 22 types that were at least 10 bp in size (Table S5). Among these, there were two types of mononucleotides (A/T), six types of dinucleotides (AG/CT, AT/AT, and AT/AT) (Table S6 & S7), two types of trinucleotides (AAT/ATT), 12 types of tetranucleotides (ACCT/AGGT, AAGG/CCTT, AAAT/ATTT, AATT/AATT, AATG/ATTC, AAAG/CTTT), and four types of pentaucleotides (AAAAG/CTTTT, AAAAT/ATTTT). The mononucleotides were the most common type of SSR detected (68%), the pentanucleotides were the most extended form of SSR, and hexanucleotides were not found in *C. sabulosa* (Fig. 4a). The number of SSRs identified in intergenic spacer regions was higher than in other locations (Fig. 4b). The LSC had the most SSRs, followed by the SSC, while the inverted repeats had the fewest (Fig. 4c).

### Oligonucleotide repeats analysis of *C. sabulosa*

**Fig. 5:**
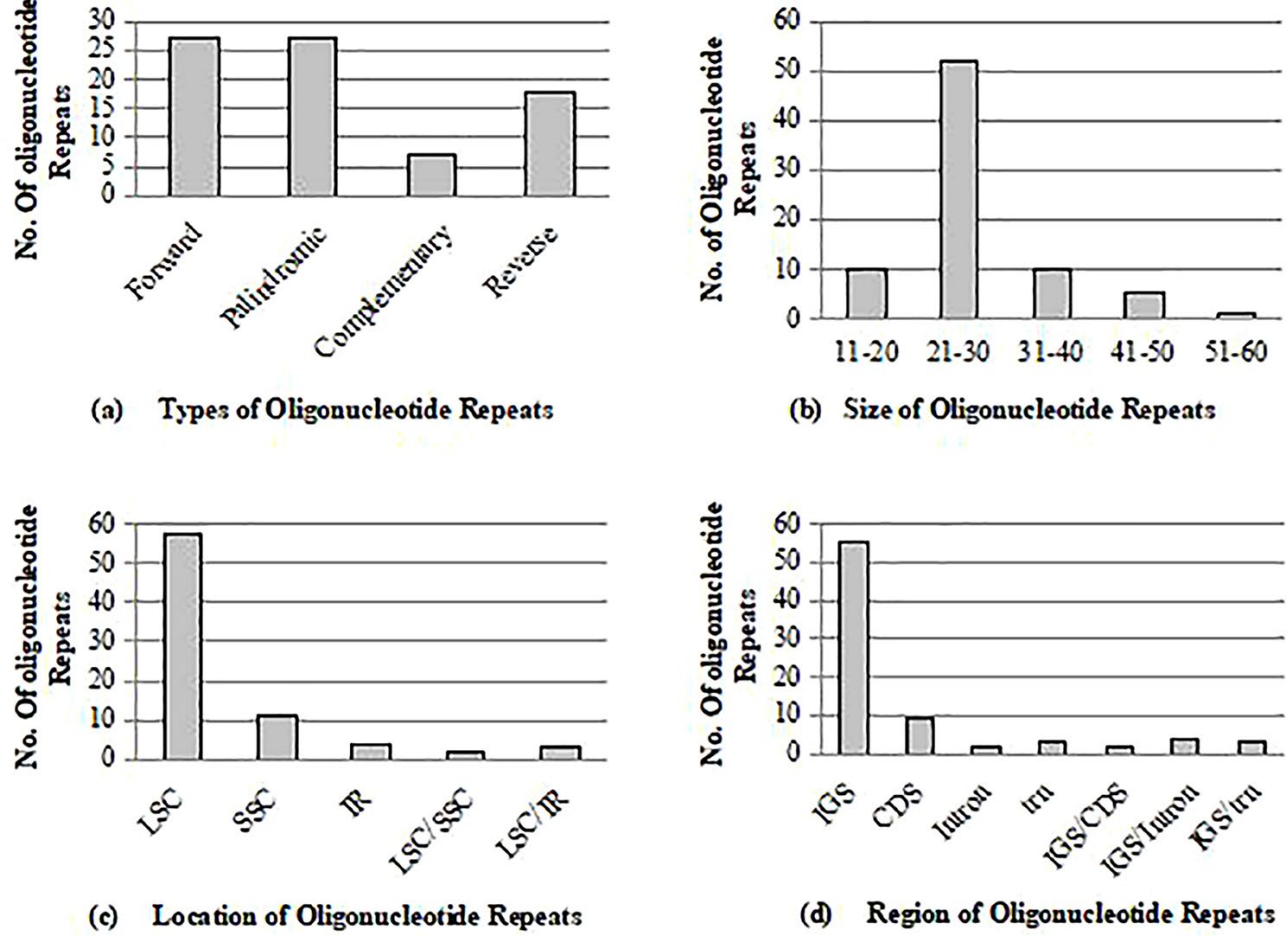
Oligonucleotide repeats analysis in *C. sabulosa*

We used the REPuter tool to identify 79 oligonucleotide repetitions, which had the following values: *P* = 27, F = 27, C = 7, and *R* = 18, as illustrated in Fig. 5. These repeats had a size ranging from 20 to 51 bp (as shown in Fig. 5b). We found that the LSC contained 57 oligonucleotide repeats, the SSC had 11, and the IRs had four. Furthermore, we discovered that IR and LSC shared three repeat layouts, with two shared by LSC/SSC and one shared by SSC/IR (as depicted in Fig. 5c). The number of oligonucleotide repetitions in intergenic spacer areas was highest (55), followed by CDS (9), transfer RNA (3), and intronic region (2). We also detected mutual repeats in the IGS/CDS (2), IGS/Intron (4), and IGS/trn (3) regions (as shown in Fig. 5d). Palindromic repeats were more frequent than other repetitions (as seen in Fig. 5a). The locations, positions, and areas of oligonucleotide repeat sequences are provided in Table S8.

### Phylogenetic analyses

**Fig. 6:**
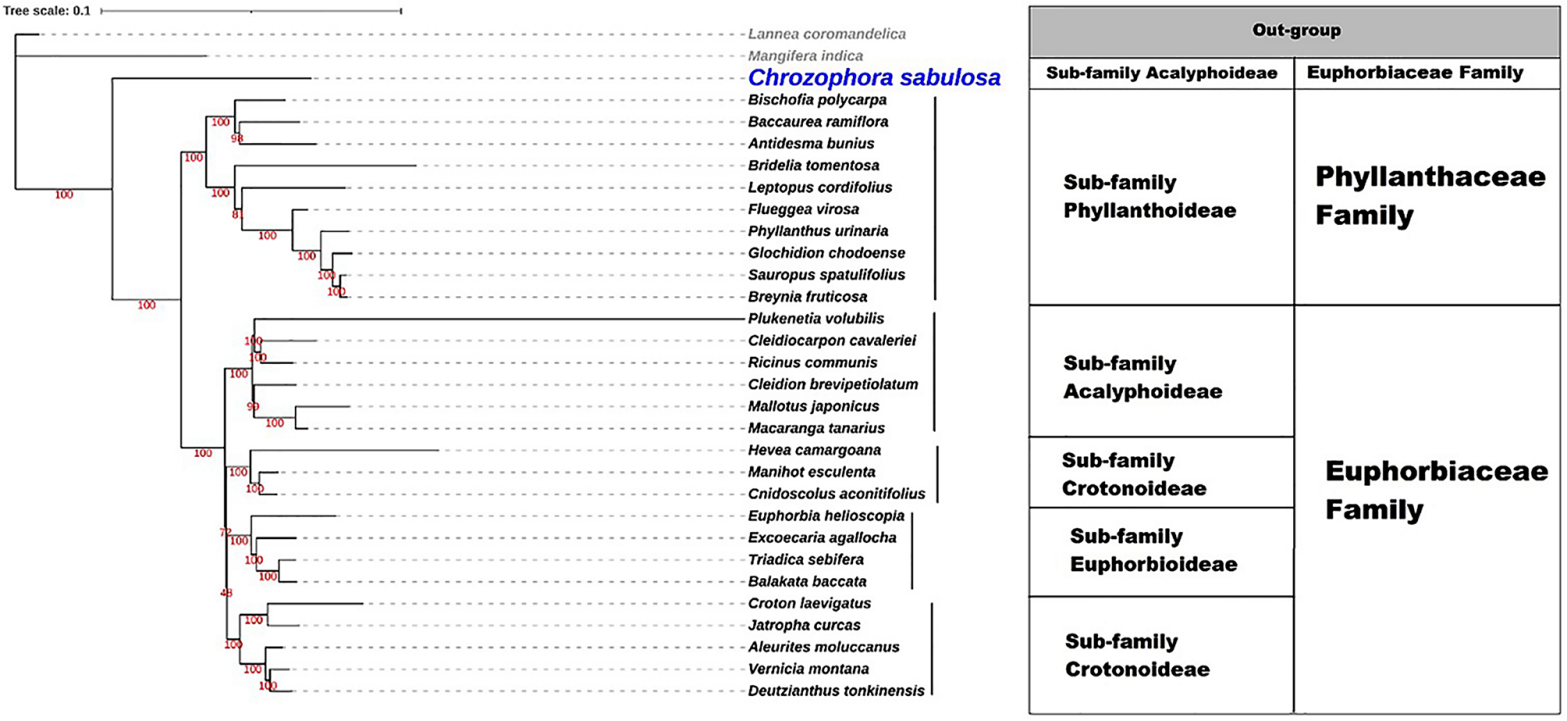
CDS-based ML tree of Euphorbiaceae and Phyllanthaceae species

We created a maximum likelihood tree using CDS data from Euphorbiaceae and Phyllanthaceae cp. genomes (Fig. 6, Table S12) to investigate the evolutionary relationship of *Chrozophora*. We constructed the tree by using C. sabulosa, 18 other genera from Euphorbiaceae, and 10 genera from Phyllanthaceae. The alignment of 31 species using MAFFT produced an 80,283 bp consensus sequence with 22,018 (22.5%) identical positions and 84.8% pair-wise identity. The tree comprised 28 nodes with bootstrap values ranging from 48 to 100. The best-fit model for this tree was GTR + F + R6. Its log-likelihood was − 471155.9363, AIC score 942465.8726, AICc score 942465.9954, and BIC score 943196.6990. The overall length of the tree was 0.9525, with internal branch lengths of 0.2132 (22% of tree length). Our analysis indicated that the *Chrozophora* genus evolved from other Euphorbiaceae members in a paraphyletic manner. The *Chrozophora* genus was closely related to the *Bischofia* genus (*Bischofia polycarpa*) of the Phyllanthaceae family. This finding suggests that the *Chrozophora* genus is more closely associated with Phyllanthaceae members than other Euphorbiaceae members. The tree also confirmed the common ancestor of both families and showed that the members of the Phyllanthaceae family share a molecular basis with members of the Euphorbiaceae family. Additionally, our analysis revealed *Chrozophora*’s unique position within the Euphorbiaceae family.

### *C. sabulosa*’s comparison with other Euphorbiaceae and phyllanthaceae species

We have selected eight plant species from two families, Euphorbiaceae and Phyllanthaceae, to compare their chloroplast genome with *C. sabulosa*. The four selected species of Euphorbiaceae are *Ricinus communis*, *Manihot esculenta*, *Jatropha curcas*, and *Euphorbia helioscopia*, while four Phyllanthaceae species are *Antidesma bunius*, *Breynia fruticosa*, *Glochidion chodoense*, and *Phyllanthus urinaria*. A detailed basic comparison is shown in Table 3. We have compared the length of the chloroplast sequence and the quadripartite structure of each species. The length of the chloroplast sequence varied from 155,630 bp (*B. fruticosa*) to 163,856 bp (*J. curcas*), and each segment of the quadripartite structure was comparable across the analyzed plastomes. *M. esculenta* and *E. helioscopia* had the highest number of genes (132), while *B. fruticosa* and *P. urinaria* had the fewest (129). The overall GC content of these cp. genomes varied from 35.4 to 36.7%, and the gene component was comparable, except for a few missing or added genes. The *infA* gene was present in *C. sabulosa* and *A. bunius* but not in the other seven species. The *rps16* gene was absent in *J. curcas* and *E. helioscopia*, while the *petD* intron was absent in *C. sabulosa*, *R. communis*, *E. helioscopia*, and *G. chodoense*. The intron of *petB* and *rpl16* was absent in *C. sabulosa*, *R. communis*, and *E. helioscopia*. The *rpl2* intron was only absent in the plastome of *C. sabulosa*. We used Geneious Prime 2021.1.1 and the MAFFT alignment of cp. genomes from the nine species to compare the relative placements of genes across the species. The 192,904-bp consensus sequence had 103,626 (54%) identical sites and 80% pair-wise identity. This analysis demonstrates that both family members have a close association, and *C. sabulosa* demonstrated a close link with both family members. These comparisons confirm the phylogenetic results and show that the chloroplast genome helps understand the evolutionary relationships within the Euphorbiaceae and Phyllanthaceae families.

**Table 3:** Described the comparison of *C. sabulosa* cp. genome with four Euphorbiaceae and four Phyllanthaceae species

Genome features	C. sabulosa	EUPHORBIACEAE				PHYLLANTHACEAE			
		Ricinus communis	Manihot esculenta	Jatropha curcas	Euphorbia helioscopia	Antidesma bunius	Breynia fruticosa	Glochidion chodoense	Phyllanthus urinaria
Genome Size (bp)	156,488	163,161	161,453	163,856	160,041	162,160	155,630	157,085	157,673
Length of LSC (bp)	87,696	89,651	89,295	91,731	88,832	89,499	85,065	85,304	85,189
Length of SSC (bp)	19,494	18,816	18,250	17,849	17,145	19,051	19,441	17,635	17,134
Length of IR (bp)	24,649	27,347	26,954	27,138	27,032	26,805	25,562	27,073	27,675
GC content %	36.5	35.7	35.9	35.4	35.9	36.4	36.7	36.7	36.5
Total No. of genes	131	131	132	130	132	131	129	130	129
Protein Coding Genes	86	86	86	85	85	87	84	85	85
No. of tRNA genes	37	37	38	37	39	38	37	37	36
No. of rRNA genes	8	8	8	8	8	8	8	8	8
Accession Number	MW541931	JF937588	NC010433	FJ695500	MN199031	ON022043	MT863745	MK056235	OL693862

### The expansion and contraction of IRs

**Fig. 7:**
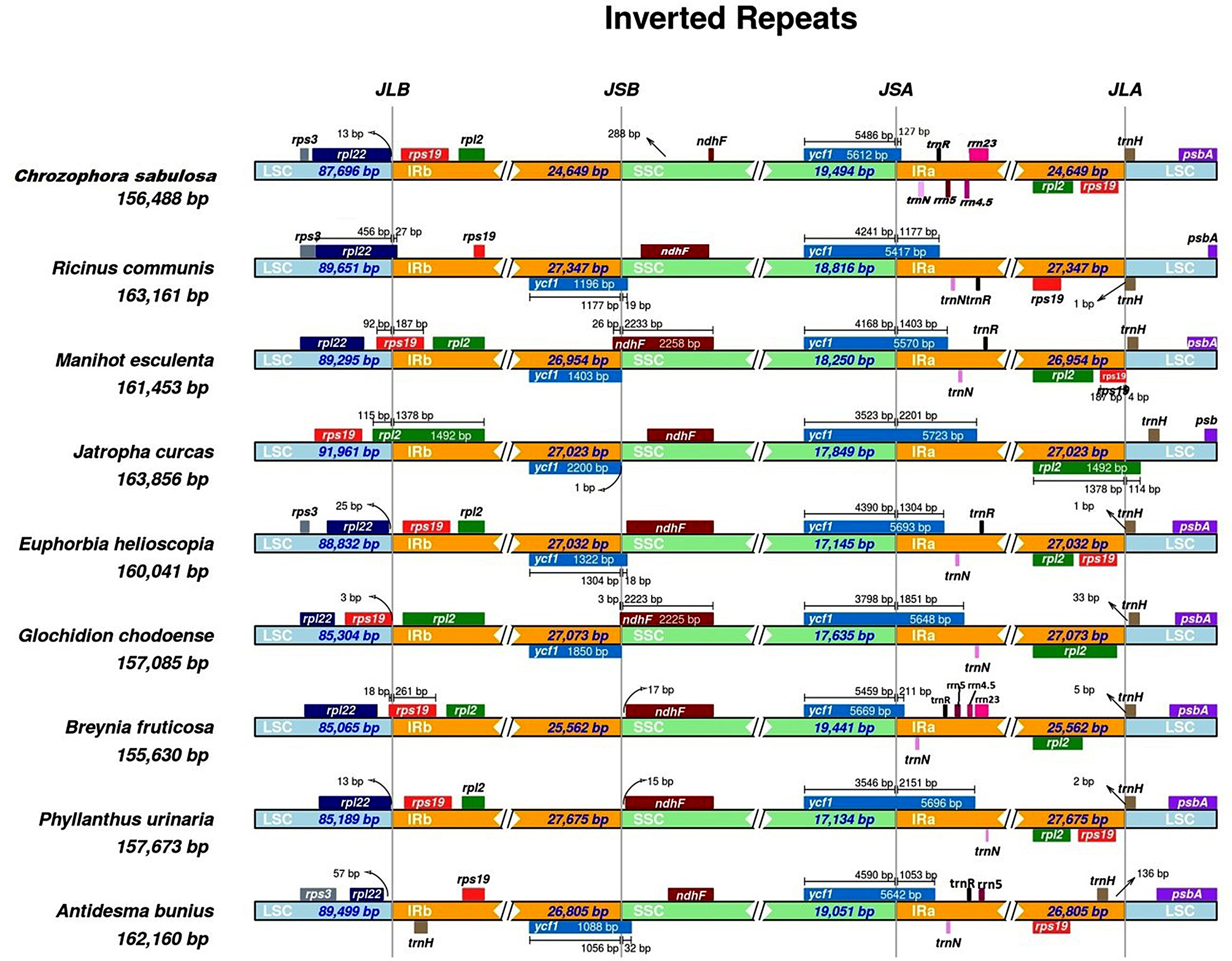
The IRSCOPE analysis of Euphorbiaceae and Phyllanthaceae species

A study was conducted on the margins of four key areas (LSC, IRB, SSC, IRA) and their surrounding genes in *C. sabulosa* and selected cp. genomes (Fig. 7). The *ycf1* gene on the JSA (SSC/IRA) junction was found to be functional in all species. However, in *R. communis*, *M. esculenta*, *J. curcas*, *E. helioscopia*, *(A) bunius*, and *G. chodoense*, a pseudo copy of *ycf1* was detected at the JSB (IRB/SSC) border, while it was absent in *C. sabulosa*, *(B) fruticosa*, and *P. urinaria*. The size and position of the *ndhF* gene varied at the JSB border. The *rps19* and *rpl2* genes were entirely in the IRs in *(C) sabulosa*, *E. helioscopia*, *A. bunius*, and *P. urinaria*, but they were found in varied locations in the other five species. The *trnH* gene was detected at the JLA (IRA/LSC) boundary in all species except *A. bunius*, which had two copies of this gene found in IR regions. The study revealed that identical genes varied in locations and sizes at every junction of the cp. genomes, indicating a variety of gene content. A thorough analysis of IR contraction and expansion is shown in Fig. 7 (IRSCOPE analysis). These findings suggest that these nine cp. genomes were slightly different due to differences in size and gene placement in these species.

### The Ka, Ks substitutions, and Ka/Ks rate

**Fig. 8:**
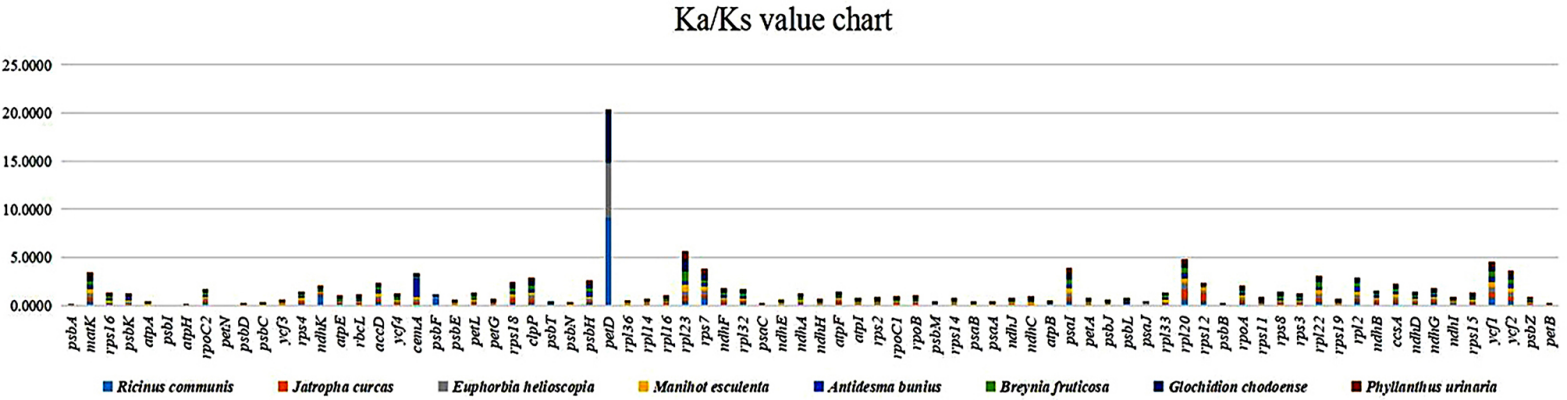
Ka/Ks values for Euphorbiaceae and Phyllanthaceae species

Pair-wise alignments of *C. sabulosa* genes were performed with eight selected cp. genomes to determine the Ka/Ks ratio (Fig. 8). All comparisons of selected genes with all species had a Ka/Ks ratio that was typically less than one. Genes for which Ka/Ks ratios were unavailable (N/A) were set to zero (See Table S10). After excluding the genes with a Ka or Ks value of zero, the average Ka/Ks ratio was 0.20, demonstrating that the genes in the cp. genome of *C. sabulosa* were subjected to significant purifying selection forces. The average Ka/Ks ratio for Euphorbiaceae species was 0.212, while it was 0.184 for the Phyllanthaceae species. The *psbI* and *petN* have zero Ka/Ks ratio in all species, making them the most stable genes among both families. Most of the genes exhibited a Ka/Ks ratio of below one in all comparisons, and their proportions were consistent, except for *petD*, *ndhK*, *cemA*, *rpl23*, and *rpl20* genes, which had challenging ratios. For instance, in *R. communis*, *E. helioscopia*, and *G. chodoense*, the Ka/Ks rate of *petD* was above one, but in the other five comparisons, it was less than one. Compared to *R. communis*, the Ka/Ks ratio of *petD* was 9.1, whereas it was only 0.03 when compared to *J. curcas*. Similarly, the Ka/Ks value of *ndhK* was 1.12 compared to *R. communis*. The Ka/Ks values of the five challenging genes are displayed in Table 4. The comprehensive Ka and Ks values and their ratios are available in Supplementary Table 9.

**Table 4:** Genes showing elusive Ka/Ks ratio for eight selected species

Sr. no	GENES	Species which pair-wise aligned with C. sabulosa	Ka/Ks
1	***petD***	*Ricinus communis*	9.085106383
		*Jatropha curcas*	0.029435163
		*Euphorbia helioscopia*	5.663157895
		*Manihot esculenta*	0.037310924
		*Antidesma bunius*	0.056277056
		*Breynia fruticosa*	0.059561966
		*Glochidion chodoense*	5.253968254
		*Phyllanthus urinaria*	0.056277056
2	***cemA***	*Ricinus communis*	0.164969982
		*Jatropha curcas*	0.188277087
		*Euphorbia helioscopia*	0.27484472
		*Manihot esculenta*	0.249648876
		*Antidesma bunius*	1.910591472
		*Breynia fruticosa*	0.208214193
		*Glochidion chodoense*	0.182541788
		*Phyllanthus urinaria*	0.195567867
3	***ndhK***	*Ricinus communis*	1.122105263
		*Jatropha curcas*	0.103290415
		*Euphorbia helioscopia*	0.109882353
		*Manihot esculenta*	0.102958937
		*Antidesma bunius*	0.122624778
		*Breynia fruticosa*	0.158464035
		*Glochidion chodoense*	0.129590208
		*Phyllanthus urinaria*	0.105896157
4	***rpl20***	*Ricinus communis*	0.606166783
		*Jatropha curcas*	1.008688097
		*Euphorbia helioscopia*	0.760762174
		*Manihot esculenta*	0.457788945
		*Antidesma bunius*	0.528846154
		*Breynia fruticosa*	0.514962037
		*Glochidion chodoense*	0.445371143
		*Phyllanthus urinaria*	0.479802513
5	***rpl23***	*Ricinus communis*	0.486597938
		*Jatropha curcas*	0.486597938
		*Euphorbia helioscopia*	0.486597938
		*Manihot esculenta*	0.7375
		*Antidesma bunius*	0.392116183
		*Breynia fruticosa*	1
		*Glochidion chodoense*	1.103950104
		*Phyllanthus urinaria*	1

### Investigating SNPs and InDel mutations analysis in *C. sabulosa*

We conducted pair-wise alignments between *C. sabulosa* and selected species of Euphorbiaceae and Phyllanthaceae families. As a result, we discovered single nucleotide polymorphisms (SNPs) and InDels mutation events in the IR, SSC, and LSC regions of *C. sabulosa*. The highest number of SNPs (18,625) was found when comparing *C. sabulosa* to *E. helioscopia*, while the lowest number was found when compared to *M. esculenta* (17,028 SNPs). All species in Table 5 had a transition-to-transversion ratio greater than one due to more transitions than transversions. The LSC and SSC regions showed a higher rate of substitutions than the IR regions. In terms of inDels, the inverted repeat regions had the fewest, while the SSC and LSC regions had the most. Pair-wise alignment of *J. curcas* resulted in the most significant number of inDels (24,369), followed by *P. urinaria* with 23,217 inDels, and *E. helioscopia* with the least (21,134). Table 6 provides an in-depth description of inDels and its relevant parameters. The similar values of InDels and SNPs observed in all the Euphorbiaceae and Phyllanthaceae species indicate their closer relationship.

**Table 5:** Ts, Tv substitutions, and Ts/Tv ratio (in IRs, SSC, and LSC) of selected Euphorbiaceae and Phyllanthaceae species

Region	Species (*C. sabulosa* as reference)	Transition substituations (Ts)		Total Ts substituations	Transversion substituaions (Tv)				Total Tv substituaions	Ts/Tv
		A/G	C/T		A/T	A/C	C/G	G/T		
Substitution Type		*R*	Y		W	M	S	K		
**Large Single Copy**	***R. communis***	3505	3806	7311	2000	1303	569	1395	5267	**1.39**
	***J. curcas***	3683	3921	7604	2350	1399	551	1424	5724	**1.33**
	***E. helioscopia***	3832	4061	7893	2512	1553	706	1624	6395	**1.23**
	***M. esculenta***	3546	3845	7391	2145	1260	560	1419	5384	**1.37**
	***A. bunius***	3723	3970	7693	2130	1371	647	1473	5621	**1.37**
	***B. fruticosa***	3697	3876	7573	2214	1344	653	1446	5657	**1.34**
	***G. chodoense***	3682	3908	7590	2234	1391	634	1470	5729	**1.32**
	***P. urinaria***	3661	3924	7585	2167	1358	647	1488	5660	**1.34**
**Inverted Repeat**	***R. communis***	273	330	603	54	113	65	112	344	**1.75**
	***J. curcas***	257	303	560	46	96	65	102	309	**1.81**
	***E. helioscopia***	295	337	632	61	118	79	113	371	**1.70**
	***M. esculenta***	288	340	628	57	106	65	115	343	**1.83**
	***A. bunius***	305	328	633	71	120	72	133	396	**1.60**
	***B. fruticosa***	308	329	637	66	110	67	124	367	**1.74**
	***G. chodoense***	298	344	642	65	107	68	138	378	**1.70**
	***P. urinaria***	306	337	643	75	119	69	139	402	**1.60**
**Small Single Copy**	***R. communis***	960	959	1919	658	367	146	433	1604	**1.20**
	***J. curcas***	913	880	1793	617	326	128	379	1450	**1.24**
	***E. helioscopia***	870	894	1764	566	374	189	441	1570	**1.12**
	***M. esculenta***	914	918	1832	577	349	123	401	1450	**1.26**
	***A. bunius***	970	927	1897	641	386	153	477	1657	**1.14**
	***B. fruticosa***	967	1004	1971	658	381	189	457	1685	**1.17**
	***G. chodoense***	861	942	1803	615	361	168	410	1554	**1.16**
	***P. urinaria***	846	891	1737	581	330	172	403	1486	**1.17**

**Table 6:** The detailed analysis of InDels, Average InDel Length, InDel Diversity K(i), InDel Diversity per site Pi(i), and alignment length in LSC, IR, and SSC regions of *C. sabulosa* by making pairwise alignment with eight selected species of Euphorbiaceae and Phyllanthaceae families

Region	Species (C. sabulosa as reference)	Alignment length	No. of InDels	InDel average Length	InDel diversity k(i)	InDel diversity per site Pi(i)
Large single copy	*R. communis*	96,729	16,111	15.857	1016.000	0.01050
	*J. curcas*	97,699	15,971	14.159	1128.000	0.01155
	*E. helioscopia*	95,542	14,556	13.693	1063.000	0.01113
	*M. esculenta*	95,837	14,703	14.030	1048.000	0.01094
	*A. bunius*	96,398	15,601	14.621	1067.000	0.01107
	*B. fruticosa*	93,881	15,001	14.867	1009.000	0.01075
	*G. chodoense*	93,909	14,818	14.527	1020.000	0.01086
	*P. urinaria*	94,125	15,365	15.427	996.000	0.01058
Inverted Repeat	*R. communis*	27,634	3272	30.868	106.000	0.00384
	*J. curcas*	28,159	4531	43.990	103.000	0.00366
	*E. helioscopia*	27,302	2923	27.838	105.000	0.00385
	*M. esculenta*	27,397	3191	28.491	112.000	0.00409
	*A. bunius*	27,284	3114	31.140	100.000	0.00367
	*B. fruticosa*	26,187	2163	19.313	112.000	0.00428
	*G. chodoense*	27,836	3950	34.052	116.000	0.00417
	*P. urinaria*	28,112	3900	34.821	112.000	0.00398
Small Single Copy	*R. communis*	20,641	2972	16.697	178.000	0.00862
	*J. curcas*	20,605	3867	22.224	174.000	0.00844
	*E. helioscopia*	20,147	3655	24.205	151.000	0.00749
	*M. esculenta*	20,614	3484	20.862	167.000	0.00810
	*A. bunius*	20,697	2849	16.096	177.000	0.00855
	*B. fruticosa*	20,749	2563	14.901	172.000	0.00829
	*G. chodoense*	20,473	3817	25.447	150.000	0.00733
	*P. urinaria*	20,290	3952	26.523	149.000	0.00734

### The nucleotide diversity and highly polymorphic loci of Euphorbiaceae and Phyllanthaceae species, with particular reference to *C. sabulosa*

**Fig. 9:**
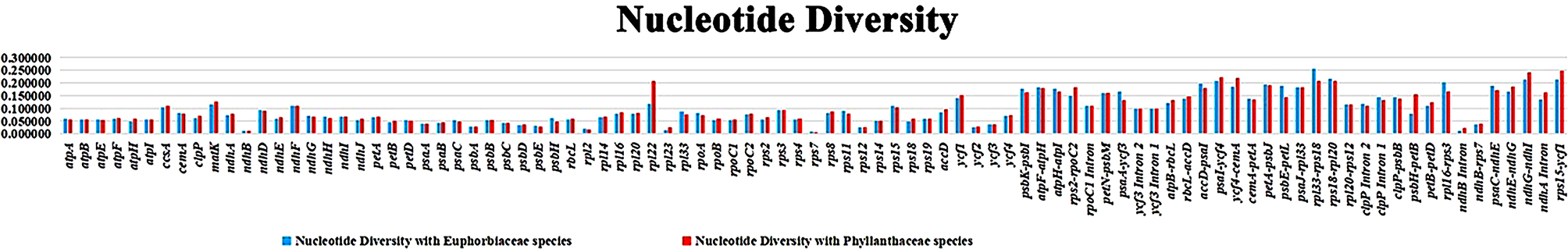
Nucleotide diversity (π) in 93 regions common in Euphorbiaceae and Phyllanthaceae family members. The π values of Euphorbiaceae members are displayed in sky blue, whereas the π values of Phyllanthaceae members are depicted in red

We conducted an independent analysis by comparing *C. sabulosa* with representatives of both Euphorbiaceae and Phyllanthaceae families. We aimed to examine the nucleotide diversity and highly polymorphic loci for *C. sabulosa* and other chosen species of these two families. We found that the average nucleotide diversity in Euphorbiaceae species was 0.0958, whereas in Phyllanthaceae species, it was 0.0981. The nucleotide diversity values varied across species and regions. In Euphorbiaceae species (Table S10), it ranged from 0.0076(*rps7*) to 0.2549(*rpl33-rps18*), whereas in Phyllanthaceae species (Table S11), it ranged from 0.0068(*rps7*) to 0.2467(*rps15-ycf1*). We observed that the average nucleotide diversity in coding areas was the lowest (Euphorbiaceae species 0.0624, Phyllanthaceae species 0.0652), followed by IGS regions (Euphorbiaceae species 0.1934, Phyllanthaceae species 0.1953), and Intronic regions (Euphorbiaceae species 0.6178, Phyllanthaceae species 0.6193). Additionally, we identified six highly polymorphic sites shared by both families (Table 7), which can be used as mutational markers to identify and classify *Chrozophora* species. Figure 9 shows the nucleotide diversity values for the 93 locations selected from both families. We found that *C. sabulosa* had identical nucleotide diversity values with both family members, indicating that it shares characteristics of both families.

**Table 7:** Six mutual highly polymorphic regions of selected Euphorbiaceae and Phyllanthaceae species

Sr. No	Region	Location	Nucleotide Diversity with Euphorbiaceae Species	Nucleotide Diversity with Phyllanthaceae Species
1	*rpl33-rps18*	IGS	0.255	0.208
2	*rps18-rpl20*	IGS	0.218	0.208
3	*rps15-ycf1*	IGS	0.214	0.247
4	*ndhG-ndhI*	IGS	0.214	0.24
5	*psaI-ycf4*	IGS	0.209	0.222
6	*petA-psbJ*	IGS	0.193	0.192

## Discussion

We are excited to present our novel findings on the chloroplast genome of *C. sabulosa*, a first-time report in the scientific community. To determine the phylogenetic position of this genus, we conducted a comprehensive comparative analysis with members of the Euphorbiaceae and Phyllanthaceae families. Specifically, we compared *C. sabulosa* to four Euphorbiaceae species (*Ricinus communis*, *Manihot esculenta*, *Jatropha curcas*, and *Euphorbia helioscopia*) and four Phyllanthaceae species (*Antidesma bunius*, *Breynia fruticosa*, *Glochidion chodoense*, and *Phyllanthus urinaria*). Our investigation covered various aspects such as cp. genome structure, gene details and their functions, GC content, intron presence or absence, amino acid frequencies, relative codon use values, RNA editing sites, SSRs, and oligonucleotide repeats. Notably, the cp. genome of *C. sabulosa* exhibits a typical quadripartite architecture and comparable structure and genomic data to other Euphorbiaceae and Phyllanthaceae species [9, 10, 22–26].

Our research on the chloroplast genome of *C. sabulosa* unveiled unique and intriguing findings. The TCG codon in the *psbL* gene of *C. sabulosa* leads to Threonine as the first amino acid, a behavior similar to that observed in the cp. genomes of other plant species such as *Indigofera* genus [27], *Spinacia oleracea* (NC_002202), *Nicotiana tabacum* (NC_001879), *Ampelopsis glandulosa* (KT831767), *Lycium barbarum* (MH032560), and *Lycium chinense* (MK040922). This underscores the conserved structure of the chloroplast genome, a feature observed in various other angiosperm lineages [17, 28–31]. Our findings also revealed that the DNA GC percentage is not uniform within the chloroplast genomic domains, with the GC concentration in the IR area being more significant than that of the other regions, likely due to the high GC concentration found in the four rRNAs in the inverted repeats [31].

Furthermore, our comparison of the cp. genome of *C. sabulosa* with other species in the Euphorbiaceae and Phyllanthaceae families yielded significant findings. The cp. genomes and gene content were similar across selected species. However, the *infA* gene was only present in *C. sabulosa* and *(A) bunius* and was missing in other Euphorbiaceae and Phyllanthaceae cp. genomes. The pseudo copy of *ycf1* was absent in *C. sabulosa*, *(B) fruticosa*, and *P. urinaria*. These findings highlight the unique gene content and organization in *(C) sabulosa* and its evolutionary implications [32–37]. We also discovered that the *petD*, *petB*, *rpl16*, and *rpl2* genes of *C. sabulosa* and some other species lacked introns, a phenomenon documented in various other angiosperms [38, 39]. The genes in which intron loss was reported earlier in other angiosperms are *rpoC2*, *atpF*, *rpl2*, *rps12*, *atpF*, *rps16*, and *clpP* [38–42]. This underscores the critical role of introns in gene expression control and their potential to boost exogenous gene expression at plant genome regions to achieve desirable agronomic features [37]. The lack of specific introns may cause changes in gene expression [37].

The codon usage bias in the cp. genome of plants is an essential evolutionary characteristic that affects the translation of mRNA, gene identification, and molecular biological investigations [32]. Some genes in plastoms have shown a bias towards specific codons, likely due to external pressure [43]. *C. sabulosa*’s cp. genome uses leucine most commonly and cysteine uncommonly. Similar findings have been reported in other cp. genomes, such as *Eruca sativa* [43], *Farsetia hamiltonii* [17], and *Nasturtium officinale* [44]. AGA codon in *C. sabulosa*’s cp. genome had the highest usage bias for Arginine. These findings suggest that codon usage significantly impacts the reshaping and translation of the cp. genome [17, 19–21, 43–47]. Our results also support earlier studies on the adaptational evolution of the large A/T concentration in chloroplast genomes, which have also shown a preference for specific codons [17, 19, 22, 23].

RNA editing is a modification after transcription, significantly impacting the sequencing and performance of related proteins and genetic material [45]. Analyzing the RNA editing sites in *C. sabulosa* could provide evolutionary insights into how RNA editing systems evolved during the evolution of plant life on Earth and which editing sites may have been maintained to carry out essential functions. Most RNA editing sites were found in the *ndhB* gene, which encodes for NADH dehydrogenase subunits. This demonstrated that a single gene could translate a wide range of protein products using RNA editing [41]. Changes at the second position of the nucleotide were more prevalent than changes at other positions among the RNA editing sites examined. RNA editing, particularly at the second codon position, can alter the encoding amino acid and the primary, secondary, and tertiary protein organization, which may be essential for their function [41, 45]. Most of the RNA editing sites were discovered in Serine codons, with the most significant transformation of Serine into Leucine, possibly increasing the hydrophobicity of the associated peptide. Our findings also supported that RNA editing sites can restore amino acid conservation, improve hydrophobicity, and impact protein architecture [41]. These findings were consistent with the fundamental properties of chloroplast gene RNA editing in higher plants [17, 41, 45].

Our study on the *C. sabulosa* cp. genome revealed that mono-nucleotide repeats were the most common and pentanucleotide was the most extended SSR type. Our results were consistent with similar studies on angiosperm species, indicating that polyadenine and polythymine repeats are more abundant in cp. genomes [17, 26, 29, 44, 51, 52]. We did not observe any hexanucleotide SSRs in the *C. sabulosa* cp. genome, which is a shared trait with *Brassica napa*, *Nasturtium officinale, Raphanus sativus*, and *Fritillaria* cp. genomes [44, 48, 49]. Single-copy regions had a higher percentage of oligonucleotide repeats than inverted repeats, confirming the reverse nature of inverted repeats [17]. The IGS had more repeats than other cp. genome regions, indicating higher susceptibility to mutations and recombination [17, 29]. Palindromic repeats were more frequent than other types of repetitions, suggesting the existence of various identical or comparable sequences, either continuous or separated by a spacer region [17, 28, 29]. Our findings were consistent with several studies conducted on angiosperms [17, 28, 29, 37, 53–55].

High-dimensional sequencing methods have made it easier to access CP genomes with vast amounts of genetic material [17, 43]. For phylogenetic research, cp. genome sequences are an excellent resource [17, 43, 53, 56, 57]. The Euphorbiaceae family is one of the most diverse angiosperm families [4, 5, 58]. However, there is conflict in sub-famil classification within the Euphorbiaceae family. Previously, based on pollen morphology, the Euphorbiaceae was classified into five sub-families, including Phyllanthoideae, Oldfieldioideae, Acalyphoideae, Crotonoideae, and Euphorbioideae [6]. Later, the Angiosperm Phylogeny Group [13, 59] separated the Phyllanthaceae from the Euphorbiaceae, giving it a separate family status. Recently, Euphorbiaceae has been divided into four subfamilies based on molecular data [59, 60]. However, this classification is also unclear due to a lack of available data on its species. Chloroplast genomes have been used to determine the phylogenetic relationships in the Euphorbiaceae family [18, 23–26]. The systematic position of *Chrozophora* was unclear until this study. The findings suggest that *Chrozophora* is closely related to the Phyllanthaceae family, which supports the historical record of the Euphorbiaceae family being the ancestor of the Phyllanthaceae family [6]. The *Chrozophora* genus is considered to be distinct from the other Euphorbiaceae family genera, indicating its paraphyletic origin. Our results also confirmed that the Euphorbiaceae family’s immense diversity, morphological divergences, variable ecological range distribution, and the scarcity of literature on numerous species make phylogenetic interpretations challenging [60]. Additionally, there is a critical need to sequence more chloroplast genomes of the Chrozophoreae tribe to clarify its position among these two families.

The cp. genome is known to be stable across different plant lineages. However, the expansion and contraction of IRs can alter the size of the cp. genome and its segments [17, 28, 53, 61, 62]. The expansion and contraction of IRs affect genes, substitutions, and genome length, ultimately determining a species’ phylogenetic position [17, 34, 63]. Previous studies have found that changes in the boundaries of the cp. genome are caused by differences in the number and location of genes at the interface of inverted repeats [17, 34]. We studied the IR regions of *C. sabulosa*, four Euphorbiaceae, and four Phyllanthaceae species. Our findings revealed both similarities and genetic variations in these plastomes. The conservation of the IR region was higher in all plastomes, while most substitutions occurred in the LSC and SSC regions. These results are consistent with similar studies on other plastid genomes [17, 28, 62–64]. Our research also showed that gene migration between single copies and inverted repeat regions causes mutation rate variations, making chloroplast genomes either conserved [17, 28, 31, 34] or widely polymorphic in gene content and structure [64–67].

The Ka/Ks ratio confirms the selective forces that have acted upon the genes during evolution. These forces can be impartial, pure, or positive depending on the Ka/Ks proportion [17, 68, 69]. We compared the cp. genome of *C. sabulosa* to eight selected species to validate our phylogenetic findings. Our analysis showed that most *C. sabulosa* genes underwent purifying selection to maintain their preserved function. Purifying selection on most chloroplast genes in the Euphorbiaceae and Phyllanthaceae family species contributed to corroborating phylogenetic results. However, a few genes showed abnormal behavior regarding Ka/Ks values, indicating *petD*, *ndhK*, *cemA*, *rpl23*, and *rpl20* in both family representatives. Our research findings are in collaboration with the previously published similar results [17, 43, 46, 70, 71]. InDels and SNPs were more frequent in the LSC section than in the inverted repeats region. The IRs had the lowest number of mutations, indicating their conserved nature over the single-copy regions [17, 28, 62–64]. The transition-to-transversion ratio was more significant than one in all selected species, indicating a higher transition rate than the transversion rate. This suggests that the species are distant and have more SNPs [17, 22]. The high Ts/Tv ratio may be due to the GC-rich composition of the chloroplast genome [61]; similarly, the nuclear genomes have already been reported to have a Ts/Tv ratio due to their GC-rich makeup [72]. The high number of InDels and SNPs (significant mutations) indicated *Chrozophora*’s unique phylogenetic position and paraphyletic evolution.

We analyzed nucleotide diversity in *C. sabulosa* and selected species from the Euphorbiaceae and Phyllanthaceae families. Results showed that IGS regions have higher rates of genetic recombination and polymorphisms than protein-coding regions. These findings confirmed that IGS regions are more susceptible to genetic recombination and polymorphisms than protein-coding regions. These outcomes also supported the conserved status of the protein-coding genes reported earlier in other plastid genomes [17, 28, 31, 34]. Nucleotide diversity is low in Euphobiaceae and Phyllanthaceae species, ranging from 0.007 to 0.24. This suggests that the plastome architecture is conserved in both families, consistent with previous studies [17, 72–76]. We identified six highly polymorphic regions shared by both families that could be used as molecular identifiers for the *Chrozophora* genus *(rpl33-rps18*, *rps18-rpl20*, *rps15-ycf1*, *ndhG-ndhI*, *psaI-ycf4*, *petA-psbJ*) all with π > 0.5. For further validation of the results of this study, more species of the *Chrozophora* genus and the Chrozophoreae tribe must be sequenced.

## Conclusion

This is the first cp. genome of *C. sabulosa*, which is also the first member of the genus and tribe reported. It has a typical quadripartite structure and gene content that is quite similar to other chloroplast genomes. Our comparative analysis with other Euphorbiaceae and Phyllanthaceae species has highlighted the conserved structure of the chloroplast genome, the non-uniform distribution of GC percentage, and the unique gene content and organization of *C. sabulosa*. Our investigation into codon usage bias and RNA editing sites has provided insights into the evolutionary characteristics of the cp. genome and the potential impact on protein organization and function. The phylogenetic analysis in this study revealed *Chrozophora*’s unique position in the Euphorbiaceae family, supporting the idea that this genus is paraphyletic. This chloroplast genome from *C. sabulosa* will be useful for the molecular characterization of related Chrozophoreae tribe species in the future. The phylogenetic data presented by this study will also aid in determining the genus’ location in Euphorbiaceae family. The highly polymorphic loci identified in this study could be used as markers for future *Chrozophora* species identification. Furthermore, it is essential to sequence its sister *Chrozophora* species to fully comprehend its phylogenetic position and evolutionary dynamics. Overall, our study provides valuable information on the chloroplast genome of *C. sabulosa* and its evolutionary implications for the scientific community.

## Materials and methods

### Collection of plant material and its sequencing

Nida Javaid and Shagufta Jabeen conducted the formal plant material identification for this study. Fresh leaves of *Chrozophora sabulosa* plants were collected from the Lesser Cholistan desert in Pakistan (28.7719699, 71.3346211) and the verification process was carried out at the Cholistan Institute of Desert Studies (CIDS) of the Islamia University Bahawalpur. The herbarium of plants was submitted to the CIDS for identification. A voucher number was issued for *Chrozophora sabulosa* Kar. & Kir. Nilkari of Euphorbiaceae which is CIDS/IUB-1601/59. For DNA extraction, the phenol-chloroform (Organic) [77] procedure was used with a few modifications. These modifications included using 1 µL 2-Mercaptoethanol and precipitating DNA with absolute ethanol following a wash with 70% ethanol. The extracted DNA was then analyzed for its quantity and quality using Nanodrop and 1% agarose gel electrophoresis. A full genome shotgun was generated by a Paired-end library of 150 bp with 350 bp insert size using Illumina Hiseq2500 at the Beijing Institute of Genomics (BIG), Beijing, China.

### Chloroplast genome assembly and genes annotation

We used FastQC software [78] to verify raw reads, and NOVOPlasty [79] to assemble the cp. genome. The LSC, SSC, and IR regions were defined manually by inspecting the sequence scaffolding. We employed GeSeq [80] (https://chlorobox.mpimp-golm.mpg.de/geseq.html) and CpGAVAS (http://www.herbalgenomics.org/cpgavas) to annotate the assembled cp. genome [81]. The annotation was manually verified using MAFFT alignment (Multiple Alignment with Fast Fourier Transform) [82] in Geneious Prime 2023.2.1 software [83]. To confirm the tRNA genes, tRNAscan-SE 1.23 program was used [84]. We determined the average sequencing coverage depth for the assembled *C. sabulosa* genome by mapping sequencing short reads to their respective de novo assembled cp. genomes using Tablet [85] and BWA [86]. We created the circular map of the cp. genome using OGDraw v1.2 (https://chlorobox.mpimp-golm.mpg.de/OGDraw.html) [87]. The *C. sabulosa* cp. genome was submitted to GenBank and assigned the accession number MW541931. We also submitted the raw data obtained in this work to Sequence Read Archive (SRA) under project number PRJNA660981.

### RNA editing site, codon usage, and amino acid frequency

We used Geneious Prime 2023.2.1 to analyze the amino acid frequency, and MEGA-X to examine Relative Synonymous Codon Usage (RSCU) in protein-coding sequences of *C. sabulosa* [88]. Additionally, we used Predictive RNA Editors for Plants Chloroplast (PREP-cp: http://prep.unl.edu/) to find RNA editing sites in 21 protein-coding genes [89].

### Detecting simple sequence repeats (SSRs) and oligonucleotide repeats

The Perl script MIcroSAtellite Identification Tools (MISA) [90] software (https://webblast.ipk-gatersleben.de/misa/) was used to identify simple sequence repeats (SSRs), with minimal repeat count of ten for mono-, five for di-, four for tri-, three tetra-, three Penta-, and three for hexanucleotides. Additionally, the REPuter program (https://bibiserv.cebitec.uni-bielefeld.de/reputer) was utilized to detect reverse (R), complementary (C), palindromic (P), and forward (F) oligonucleotide repeats with an edit distance of two, and minimum repeat size of 10 bp. The maximum calculated repeat was set to 100 [91].

### Phylogenetic analysis

The National Center for Biotechnology Information (NCBI) was used to select 18 species plastomes from the Euphorbiaceae family for phylogenetic analysis (Table S12). These species represent 11 tribes and three subfamilies of the Euphorbiaceae, including Acalyphoideae, Crotonoideae, and Euphorbioideae. Additionally, 10 species of the Phyllanthaceae family and two out-groups (*Mangifera indica* and *Lannea coromandelica*) from the Anacardiaceae family were chosen for analysis. In total, 31 species were included in the phylogenetic tree. The protein-coding sequences for each species were extracted and concatenated using Geneious Prime 2021.1.1. The sequences were then aligned using MAFFT in Geneious Prime 2023.2.1. The maximum likelihood tree was constructed online in Galaxy (https://usegalaxy.org) using IQ-TREE [92] and 1000 bootstrap replications with Ultrafast bootstrap settings [93]. The best-fit model was selected according to the Akaike information criterion (AIC) [94]. We completed the tree display using iTOL (https://itol.embl.de/#) [95].

### Comparative analyses with *C. Sabulosa*

The cp. genomes of four species from the Euphorbiaceae family (*Ricinus communis*, *Manihot esculenta*, *Jatropha curcas*, and *Euphorbia helioscopia*) and four species from the Phyllanthaceae familys (*Antidesma bunius*, *Breynia fruticosa*, *Glochidion chodoense*, and *Phyllanthus urinaria*) were compared to that of *C. sabulosa*. This was done using phylogenetic analysis outcomes. The Geneious Prime 2023.2.1 was used to perform a basic comparison of the plastomes. IRscope (https://irscope.shinyapps.io/irapp/) was used to observe IR contraction and expansion in the LSC/IRB/SSC/IRA junctions among these selected species [96]. Pairwise comparisons of the 78 protein-coding genes common in *C. sabulosa* and the eight selected species were performed to estimate synonymous (Ks) and non-synonymous (Ka) substitution rates. *C. sabulosa* was used as the reference member to make pairwise alignments with every gene of the selected species. Firstly, MAFFT in Geneious Prime 2023.2.1 software was used to perform 624 pairwise alignments of the identified genes among species [82, 83], and then DnaSP [97] was employed to examine Ka and Ks substitutions. Geneious Prime 2023.2.1 was used to calculate the number, coordinate placements, and types of substitutions (transition and transversion). DnaSP [97] was used to find InDels mutations for each part of the pairwise aligned cp. genomes. The alignment length, inDel average length, k(i) inDel diversity, and Pi(i) inDel diversity per site were also calculated.

### Nucleotide diversity to determine highly polymorphic loci

We analyzed to compare Nucleotide diversity (π) values among representatives of both families along with *C. sabulosa.* We extracted a total of 837 regions, which included 59 CDS genes, 27 IGS regions, and seven intronic regions that were common in all species. To create multiple alignments of 93 locations of *C. sabulosa* with members of both families separately, we used MAFFT [82]. We only selected sequences that were longer than 200 base pairs [98]. To calculate the Nucleotide diversity (π), we used DnaSP [97]. We identified six highly polymorphic loci that had greater nucleotide diversity to compare among the selected species [98].

### Electronic supplementary material

Below is the link to the electronic supplementary material.

Supplementary Material 1

## Data Availability

The datasets generated and/or analyzed during the current study are available in the NCBI repository, https://www.ncbi.nlm.nih.gov/nuccore/2419468502 ACCESSION MW541931. Raw data submitted Sequence Read Archive (SRA) under the project number PRJNA660981. Further data is present in the manuscript. For more in-depth details, there is a supplementary file.
