# Supplementary material for: The chloroplast genome of Chrozophora sabulosa Kar. & Kir. and its exploration in the evolutionary position uncertainty of genus Chrozophora
Source: BMC Genomics. 2024 Jun 14;25:597. doi: 10.1186/s12864-024-10366-3 (PMC11177538; doi:10.1186/s12864-024-10366-3)
Supplement: Supplementary file 1 — Supplementary Material 1 [file 12864_2024_10366_MOESM1_ESM.docx]

**The chloroplast genome of *Chrozophora sabulosa* Kar. & Kir. and its exploration in the evolutionary position uncertainty of Genus *Chrozophora*.**

Nida Javaid^3^, Musarrat Ramzan^3*^, Shagufta Jabeen^3^, Yanjun Du^1^, Muhammad Anwar^1,2*^, SONG Xiqiang^1,2*^

^1^School of Tropical Agriculture and Forestry, Hainan University, Haikou, P. R China

^2^Key Laboratory of Genetic and Germplasm Innovation of Tropical Special Forest Trees and Ornamental Plants (Hainan University) Ministry of Education, Hainan University, Haikou, P. R China

^3^The Islamia University Bahawalpur, Pakistan

Corresponding authors: Muhammad Anwar ([anwar_uaar@yahoo.com](mailto:anwar_uaar@yahoo.com))

Address: School of Tropical Agriculture and Forestry, Hainan University, Haikou, People’s Republic of China

***Supplementary Material***

1. **Supplementary tables**

**Supplementary table 1:** Genes containing introns and their length in *Chrozophora sabulosa (C. sabulosa).*

| **Genes** | **Strand** | **Gene length** | | **Length** | **Exon I** | **Intron I** | **Exon II** | **Intron II** | **Exon III** |
| --- | --- | --- | --- | --- | --- | --- | --- | --- | --- |
|  |  | **Start** | **End** |  |  |  |  |  |  |
| *clpP* | reverse | 73595 | 75668 | 588 | 69 | 881 | 291 | 602 | 228 |
| *ycf3* | reverse | 43726 | 45777 | 510 | 107 | 778 | 228 | 767 | 155 |
| *rpoC1* | reverse | 20437 | 23279 | 1965 | 430 | 800 | 1603 |  |  |
| *ndhB* | reverse | 97957 | 100157 | 1533 | 777 | 668 | 756 |  |  |
| *ndhB* | forward | 144028 | 146228 | 1533 | 777 | 668 | 756 |  |  |
| *ndhA* | reverse | 122305 | 124469 | 1092 | 552 | 1073 | 540 |  |  |
| *atpF* | reverse | 11458 | 12802 | 555 | 144 | 790 | 411 |  |  |
| *rps16* | reverse | 4701 | 5825 | 198 | 36 | 858 | 231 |  |  |
| *rps12* | mixed | 73319 | 143184 | 372 | 114 | 545 | 231 |  | 27 |
| *rps12* | reverse | 73319 | 101803 | 372 | 114 | 545 | 231 |  | 27 |
| *trnH-GUG* | reverse | 1 | 156488 | 74 | 72 | 0 | 2 |  |  |
| *trnK-UUU* | reverse | 1699 | 4248 | 72 | 38 | 2478 | 36 |  |  |
| *trnS-CGA* | forward | 8717 | 9498 | 91 | 31 | 691 | 60 |  |  |
| *trnC-ACA* | reverse | 53852 | 54529 | 95 | 39 | 583 | 56 |  |  |
| *trnL-UAA* | forward | 49173 | 49671 | 85 | 35 | 414 | 50 |  |  |

**Supplementary table 2:** Frequency of Amino acid in *C. sabulosa*, * shows the stop codon.

| **Codon** | **A.A** | **%** | **Frequency** | **Codon** | **A.A** | **%** | **Frequency** |
| --- | --- | --- | --- | --- | --- | --- | --- |
| GCA | A | 29.00% | 412 | CCA | P | 29.60% | 322 |
| GCC | A | 16.00% | 227 | CCC | P | 18.80% | 205 |
| GCG | A | 10.90% | 155 | CCG | P | 12.70% | 138 |
| GCT | A | 44.20% | 629 | CCT | P | 38.90% | 424 |
| TGC | C | 22.80% | 69 | CAA | Q | 78.80% | 747 |
| TGT | C | 77.20% | 233 | CAG | Q | 21.20% | 201 |
| GAC | D | 19.30% | 208 | AGA | R | 29.80% | 481 |
| GAT | D | 80.70% | 868 | AGG | R | 12.10% | 195 |
| GAA | E | 76.20% | 1,056 | CGA | R | 22.40% | 362 |
| GAG | E | 23.80% | 330 | CGC | R | 6.30% | 101 |
| TTC | F | 34.20% | 526 | CGG | R | 7.60% | 122 |
| TTT | F | 65.80% | 1,010 | CGT | R | 21.90% | 353 |
| GGA | G | 40.10% | 717 | AGC | S | 5.50% | 111 |
| GGC | G | 10.60% | 189 | AGT | S | 20.70% | 416 |
| GGG | G | 17.10% | 306 | TCA | S | 20.70% | 417 |
| GGT | G | 32.20% | 576 | TCC | S | 15.00% | 302 |
| CAC | H | 26.30% | 164 | TCG | S | 8.70% | 175 |
| CAT | H | 73.70% | 460 | TCT | S | 29.40% | 593 |
| ATA | I | 31.60% | 719 | ACA | T | 31.80% | 421 |
| ATC | I | 18.80% | 428 | ACC | T | 18.20% | 240 |
| ATT | I | 49.60% | 1,130 | ACG | T | 10.40% | 138 |
| AAA | K | 75.40% | 1,097 | ACT | T | 39.60% | 523 |
| AAG | K | 24.60% | 358 | GTA | V | 38.70% | 539 |
| CTA | L | 14.20% | 395 | GTC | V | 10.90% | 152 |
| CTC | L | 7.00% | 194 | GTG | V | 13.40% | 186 |
| CTG | L | 6.40% | 178 | GTT | V | 37.00% | 516 |
| CTT | L | 21.60% | 602 | TGG | W | 100% | 467 |
| TTA | L | 31.00% | 864 | TAC | Y | 18.90% | 186 |
| TTG | L | 19.90% | 555 | TAT | Y | 81.10% | 799 |
| ATG | M | 99.70% | 619 | TAA | * | 57.00% | 49 |
| TTG | M | 0.30% | 2 | TAG | * | 22.10% | 19 |
| AAC | N | 22.00% | 284 | TGA | * | 20.90% | 18 |
| AAT | N | 78.00% | 1,009 |  |  |  |  |

**Supplementary Table 3:** Frequency of Amino acid and relative synonymous codon usage in *C. sabulosa*. * shows the stop codons.

| **Codon** | **AA** | **Freq of A.Acid** | **RSCU** | **Codon** | **AA** | **Freq of A.Acid** | **RSCU** |
| --- | --- | --- | --- | --- | --- | --- | --- |
| UUU | F | 2385 | 1.21 | UAU | Y | 1442 | 1.36 |
| UUC | F | 1568 | 0.79 | UAC | Y | 677 | 0.64 |
| UUA | L | 1214 | 1.36 | UAA | * | 1229 | 1.27 |
| UUG | L | 1198 | 1.34 | UAG | * | 756 | 0.78 |
| CUU | L | 1027 | 1.15 | CAU | H | 913 | 1.39 |
| CUC | L | 630 | 0.71 | CAC | H | 402 | 0.61 |
| CUA | L | 811 | 0.91 | CAA | Q | 1037 | 1.39 |
| CUG | L | 478 | 0.54 | CAG | Q | 453 | 0.61 |
| AUU | I | 1904 | 1.25 | AAU | N | 1930 | 1.44 |
| AUC | I | 1060 | 0.69 | AAC | N | 755 | 0.56 |
| AUA | I | 1620 | 1.06 | AAA | K | 2343 | 1.41 |
| AUG | M | 830 | 1 | AAG | K | 991 | 0.59 |
| GUU | V | 731 | 1.28 | GAU | D | 1042 | 1.44 |
| GUC | V | 420 | 0.74 | GAC | D | 404 | 0.56 |
| GUA | V | 722 | 1.27 | GAA | E | 1327 | 1.36 |
| GUG | V | 406 | 0.71 | GAG | E | 622 | 0.64 |
| UCU | S | 1167 | 1.45 | UGU | C | 646 | 1.18 |
| UCC | S | 922 | 1.15 | UGC | C | 453 | 0.82 |
| UCA | S | 931 | 1.16 | UGA | * | 925 | 0.95 |
| UCG | S | 629 | 0.78 | UGG | W | 681 | 1 |
| CCU | P | 592 | 1.01 | CGU | R | 341 | 0.65 |
| CCC | P | 590 | 1 | CGC | R | 231 | 0.44 |
| CCA | P | 757 | 1.29 | CGA | R | 537 | 1.03 |
| CCG | P | 414 | 0.7 | CGG | R | 372 | 0.71 |
| ACU | T | 651 | 1.17 | AGU | S | 720 | 0.89 |
| ACC | T | 573 | 1.03 | AGC | S | 462 | 0.57 |
| ACA | T | 643 | 1.16 | AGA | R | 1066 | 2.05 |
| ACG | T | 358 | 0.64 | AGG | R | 580 | 1.11 |
| GCU | A | 458 | 1.27 | GGU | G | 536 | 0.99 |
| GCC | A | 349 | 0.97 | GGC | G | 342 | 0.63 |
| GCA | A | 391 | 1.09 | GGA | G | 750 | 1.39 |
| GCG | A | 241 | 0.67 | GGG | G | 527 | 0.98 |

**Supplementary table 4**: RNA editing sites in *C. sabulosa.*

| **GENE NAME** | **Nt Pos** | **AA Pos** | **Align Col** | **Effect** | **Score** |
| --- | --- | --- | --- | --- | --- |
| ***accD*** | 332 | 111 | 118 | CCG (P) => CTG (L) | 1 |
|  | 803 | 268 | 288 | CCG (P) => CTG (L) | 0.8 |
|  | 1387 | 463 | 495 | CTC (L) => TTC (F) | 0.8 |
| ***atpA*** | 914 | 305 | 305 | TCA (S) => TTA (L) | 1 |
|  | 1270 | 424 | 424 | CCC (P) => TCC (S) | 1 |
| ***atpB*** | 419 | 140 | 140 | ACA (T) => ATA (I) | 0.86 |
| ***clpP*** | 556 | 186 | 187 | CAT (H) => TAT (Y) | 1 |
| ***matK*** | 1496 | 499 | 514 | TCA (S) => TTA (L) | 1 |
| ***ndhA*** | 341 | 114 | 114 | TCA (S) => TTA (L) | 1 |
|  | 566 | 189 | 189 | TCA (S) => TTA (L) | 1 |
|  | 1073 | 358 | 358 | TCC (S) => TTC (F) | 1 |
| ***ndhB*** | 149 | 50 | 50 | TCA (S) => TTA (L) | 1 |
|  | 467 | 156 | 156 | CCA (P) => CTA (L) | 1 |
|  | 586 | 196 | 196 | CAT (H) => TAT (Y) | 1 |
|  | 611 | 204 | 204 | TCA (S) => TTA (L) | 0.8 |
|  | 737 | 246 | 246 | CCA (P) => CTA (L) | 1 |
|  | 746 | 249 | 249 | TCT (S) => TTT (F) | 1 |
|  | 830 | 277 | 277 | TCA (S) => TTA (L) | 1 |
|  | 836 | 279 | 279 | TCA (S) => TTA (L) | 1 |
|  | 1481 | 494 | 494 | CCA (P) => CTA (L) | 1 |
| ***ndhD*** | 170 | 57 | 57 | ACA (T) => ATA (I) | 1 |
|  | 527 | 176 | 176 | TCA (S) => TTA (L) | 1 |
|  | 781 | 261 | 261 | CTC (L) => TTC (F) | 0.8 |
|  | 1022 | 341 | 341 | TCA (S) => TTA (L) | 1 |
|  | 1031 | 344 | 344 | CCC (P) => CTC (L) | 1 |
|  | 1442 | 481 | 481 | TCA (S) => TTA (L) | 0.8 |
|  | 1454 | 485 | 485 | TCA (S) => TTA (L) | 0.8 |
| ***ndhF*** | 290 | 97 | 97 | TCA (S) => TTA (L) | 1 |
|  | 586 | 196 | 196 | CTT (L) => TTT (F) | 0.8 |
|  | 890 | 297 | 297 | ACA (T) => ATA (I) | 1 |
| ***ndhG*** | 166 | 56 | 56 | CAT (H) => TAT (Y) | 0.8 |
|  | 250 | 84 | 84 | CCA (P) => TCA (S) | 1 |
|  | 314 | 105 | 105 | ACA (T) => ATA (I) | 0.8 |
| ***petB*** | 259 | 87 | 140 | CGG (R) => TGG (W) | 1 |
| ***psbF*** | 77 | 26 | 26 | TCT (S) => TTT (F) | 1 |
| ***psbL*** | 2 | 1 | 1 | ACG (T) => ATG (M) | 1 |
| ***rpl20*** | 370 | 124 | 124 | CCC (P) => TCC (S) | 1 |
| ***rpoA*** | 757 | 253 | 253 | CTT (L) => TTT (F) | 0.86 |
|  | 934 | 312 | 314 | CTT (L) => TTT (F) | 1 |
| ***rpoB*** | 473 | 158 | 159 | TCA (S) => TTA (L) | 0.86 |
|  | 551 | 184 | 185 | TCA (S) => TTA (L) | 1 |
|  | 566 | 189 | 190 | TCG (S) => TTG (L) | 1 |
|  | 2426 | 809 | 827 | TCA (S) => TTA (L) | 0.86 |
| ***rpoC1*** | 551 | 184 | 217 | ACT (T) => ATT (I) | 0.86 |
| ***rpoC2*** | 1505 | 502 | 521 | ACG (T) => ATG (M) | 0.86 |
|  | 2269 | 757 | 952 | CGG (R) => TGG (W) | 1 |
|  | 3118 | 1040 | 1259 | CTT (L) => TTT (F) | 0.86 |
| ***rps2*** | 248 | 83 | 83 | TCA (S) => TTA (L) | 1 |
| ***rps8*** | 143 | 48 | 48 | GCG (A) => GTG (V) | 1 |
| ***rps14*** | 80 | 27 | 27 | TCA (S) => TTA (L) | 1 |

**Supplementary table 5**: Simple sequence repeats types, size, and location in *C. sabulosa.*

| **SSR nr.** | **SSR type** | **SSR** | **Size** | **Start** | **End** | **Region** | **Locus** | **Location** |
| --- | --- | --- | --- | --- | --- | --- | --- | --- |
| 1 | c | (ATAA)4(T)10(A)12 | 107 | 213 | 319 | LSC | IGS | *trnH-GUG-psbA* |
| 2 | p2 | (AT)5 | 10 | 2012 | 2021 | LSC | CDS | *matK* |
| 3 | p1 | (A)15 | 15 | 3498 | 3512 | LSC | Intron | *trnK-UUU* |
| 4 | p1 | (A)11 | 11 | 4292 | 4302 | LSC | IGS | *trnK-UUU-rps16* |
| 5 | p1 | (T)11 | 11 | 4473 | 4483 | LSC | IGS | *trnK-UUU-rps16* |
| 6 | p1 | (T)11 | 11 | 6690 | 6700 | LSC | IGS | *trnQ-UUG-psbK* |
| 7 | p1 | (A)13 | 13 | 7360 | 7372 | LSC | IGS | *psbK-psbI* |
| 8 | p2 | (AT)5 | 10 | 7959 | 7968 | LSC | IGS | *trnS-GCU-trnS-CGA* |
| 9 | c* | (TATT)3(A)11(AAT)4* | 114 | 8158 | 8271 | LSC | IGS | *trnS-GCU-trnS-CGA* |
| 10 | p1 | (A)11 | 11 | 8514 | 8524 | LSC | IGS | *trnS-GCU-trnS-CGA* |
| 11 | p4 | (AATA)3 | 12 | 9597 | 9608 | LSC | IGS | *trnS-CGA-trnR-UCU* |
| 12 | p1 | (T)12 | 12 | 12136 | 12147 | LSC | Intron | *atpF* |
| 13 | p1 | (T)12 | 12 | 12916 | 12927 | LSC | IGS | *atpF-atpH* |
| 14 | p4 | (GGAA)3 | 12 | 13065 | 13076 | LSC | IGS | *atpF-atpH* |
| 15 | p1 | (A)10 | 10 | 13510 | 13519 | LSC | IGS | *atpH-atpI* |
| 16 | c* | (CT)4(C<T>)(T)12 | 22 | 15910 | 15931 | LSC | IGS | *rps2-rpoC2* |
| 17 | p1 | (T)10 | 10 | 16052 | 16061 | LSC | IGS | *rps2-rpoC2* |
| 18 | p1 | (T)11 | 11 | 18131 | 18141 | LSC | CDS | *rpoC2* |
| 19 | p2 | (TA)5 | 10 | 19480 | 19489 | LSC | CDS | *rpoC2* |
| 20 | p1 | (A)13 | 13 | 22603 | 22615 | LSC | Intron | *rpoC1* |
| 21 | p1 | (T)10 | 10 | 25873 | 25882 | LSC | CDS | *rpoB* |
| 22 | p1 | (T)10 | 10 | 27210 | 27219 | LSC | IGS | *rpoB-trnC-GCA* |
| 23 | p1 | (A)10 | 10 | 27998 | 28007 | LSC | IGS | *trnC-GCA-petN* |
| 24 | p1 | (A)11 | 11 | 28960 | 28970 | LSC | IGS | *petN-psbM* |
| 25 | p1 | (A)15 | 15 | 29625 | 29639 | LSC | IGS | *psbM-trnD-GUC* |
| 26 | c | (T)10(TTAA)3 | 68 | 29929 | 29996 | LSC | IGS | *psbM-trnD-GUC* |
| 27 | p2 | (TA)5 | 10 | 31116 | 31125 | LSC | IGS | *trnD-GUC-trnY-GUA* |
| 28 | p1 | (T)10 | 10 | 31682 | 31691 | LSC | IGS | *trnE-UUC-trnT-GGU* |
| 29 | p2 | (AT)5 | 10 | 31990 | 31999 | LSC | IGS | *trnE-UUC-trnT-GGU* |
| 30 | p4 | (TCTT)3 | 12 | 33148 | 33159 | LSC | IGS | *trnT-GGU-psbD* |
| 31 | p5 | (TTTTC)3 | 15 | 36165 | 36179 | LSC | IGS | *psbC-trnS-UGA* |
| 32 | p1 | (T)11 | 11 | 36643 | 36653 | LSC | IGS | *trnS-UGA-psbZ* |
| 33 | p1 | (A)11 | 11 | 46430 | 46440 | LSC | IGS | *ycf3-trnS-GGA* |
| 34 | p1 | (A)10 | 10 | 46582 | 46591 | LSC | IGS | *ycf3-trnS-GGA* |
| 35 | p2 | (TA)6 | 12 | 47989 | 48000 | LSC | IGS | *rps4-trnT-UGU* |
| 36 | p5 | (AATAA)3 | 15 | 48245 | 48259 | LSC | IGS | *rps4-trnT-UGU* |
| 37 | p1 | (A)12 | 12 | 49425 | 49436 | LSC | Intron | *trnL-UAA* |
| 38 | p1 | (T)11 | 11 | 49755 | 49765 | LSC | IGS | *trnL-UAA-trnF-GAA* |
| 39 | p1 | (T)10 | 10 | 50690 | 50699 | LSC | IGS | *trnF-GAA-ndhJ* |
| 40 | p1 | (A)13 | 13 | 51473 | 51485 | LSC | IGS | *ndhJ-ndhK* |
| 41 | p1 | (T)11 | 11 | 52200 | 52210 | LSC | CDS | *ndhK* |
| 42 | p1 | (T)10 | 10 | 54901 | 54910 | LSC | IGS | *trnM-CAU-atpE* |
| 43 | p1 | (T)10 | 10 | 56911 | 56920 | LSC | CDS | *atpB* |
| 44 | p1 | (T)11 | 11 | 61754 | 61764 | LSC | IGS | *accD-psaI* |
| 45 | p4 | (AATT)3 | 12 | 61931 | 61942 | LSC | IGS | *accD-psaI* |
| 46 | p4 | (AAAT)3 | 12 | 63320 | 63331 | LSC | IGS | *ycf4-cemA* |
| 47 | p1 | (T)10 | 10 | 63753 | 63762 | LSC | IGS | *ycf4-cemA* |
| 48 | p1 | (A)11 | 11 | 63952 | 63962 | LSC | IGS/CDS | *ycf4-cemA* |
| 49 | p1 | (T)12 | 12 | 66382 | 66393 | LSC | IGS | *petA-psbJ* |
| 50 | p1 | (T)10 | 10 | 68299 | 68308 | LSC | IGS | *psbE-petL* |
| 51 | p1 | (T)11 | 11 | 68664 | 68674 | LSC | IGS | *psbE-petL* |
| 52 | p4 | (TGAA)3 | 12 | 69111 | 69122 | LSC | IGS | *petL-petG* |
| 53 | p1 | (T)13 | 13 | 69248 | 69260 | LSC | IGS | *petL-petG* |
| 54 | p1 | (A)20 | 20 | 70502 | 70521 | LSC | IGS | *petG-trnW-CCA* |
| 55 | p1 | (A)10 | 10 | 71280 | 71289 | LSC | IGS | *rpl33-rps18* |
| 56 | p4 | (ATAA)3 | 12 | 71848 | 71859 | LSC | IGS/CDS | *rps18-rpl20* |
| 57 | p1 | (T)10 | 10 | 72063 | 72072 | LSC | IGS | *rps18-rpl20* |
| 58 | p1 | (T)10 | 10 | 73187 | 73196 | LSC | IGS | *rpl20-rps12* |
| 59 | p1 | (A)11 | 11 | 74958 | 74968 | LSC | Intron | *clpP* |
| 60 | p4 | (TTTC)3 | 12 | 78554 | 78565 | LSC | IGS | *psbH-petB* |
| 61 | p1 | (A)13 | 13 | 79072 | 79084 | LSC | IGS | *psbH-petB* |
| 62 | p1 | (A)10 | 10 | 80255 | 80264 | LSC | IGS | *petB-petD* |
| 63 | p2 | (AT)6 | 12 | 80703 | 80714 | LSC | IGS | *petB-petD* |
| 64 | p1 | (T)10 | 10 | 81867 | 81876 | LSC | CDS | *rpoA* |
| 65 | p1 | (T)10 | 10 | 83822 | 83831 | LSC | IGS | *infA-rps8* |
| 66 | c | (AT)5(T)17(A)10 | 98 | 85608 | 85705 | LSC | IGS | *rpl16-rps3* |
| 67 | p1 | (T)11 | 11 | 88047 | 88057 | IR | IGS | *rps19-rpl2* |
| 68 | p2 | (TA)5 | 10 | 97558 | 97567 | IR | IGS | *trnL-CAA-ndhB* |
| 69 | p1 | (T)13 | 13 | 102967 | 102979 | IR | IGS | *rps12-trnV-GAC* |
| 70 | p4 | (AGGT)3 | 12 | 109176 | 109187 | IR | rrn | *rrn23* |
| 71 | c | (AT)5(T)11 | 34 | 112402 | 112435 | SSC | IGS | *trnN-GUU-ndhF* |
| 72 | p3 | (ATT)4 | 12 | 113405 | 113416 | SSC | CDS | *ndhF* |
| 73 | p1 | (A)10 | 10 | 116088 | 116097 | SSC | IGS | *rpl32-trnL-UAG* |
| 74 | p1 | (T)13 | 13 | 116665 | 116677 | SSC | IGS | *trnL-UAG-ccsA* |
| 75 | p1 | (A)13 | 13 | 120222 | 120234 | SSC | IGS | *psaC-ndhE* |
| 76 | p1 | (T)14 | 14 | 123256 | 123269 | SSC | Intron | *ndhA* |
| 77 | p1 | (A)14 | 14 | 126183 | 126196 | SSC | IGS | *rps15-ycf1* |
| 78 | p4 | (TAAT)3 | 12 | 128002 | 128013 | SSC | CDS | *ycf1* |
| 79 | p1 | (T)10 | 10 | 128538 | 128547 | SSC | CDS | *ycf1* |
| 80 | p1 | (T)13 | 13 | 130981 | 130993 | SSC | CDS | *ycf1* |
| 81 | p4 | (CTAC)3 | 12 | 134996 | 135007 | IR | rrn | *rrn23* |
| 82 | p1 | (A)13 | 13 | 141206 | 141218 | IR | IGS | *trnV-GAC-rps12* |
| 83 | p2 | (AT)5 | 10 | 146617 | 146626 | IR | IGS | *rpl2-rps19* |
| 84 | p1 | (A)11 | 11 | 156128 | 156138 | IR | IGS | *ndhB-trnL-CAA* |

**Supplementary table 6**: Types and number of simple sequence repeats motifs in *C. sabulosa.*

| **Repeats** | **3** | **4** | **5** | **6** | **7** | **8** | **9** | **10** | **11** | **12** | **13** | **14** | **15** | **16** | **17** | **18** | **19** | **20** | **Total** |
| --- | --- | --- | --- | --- | --- | --- | --- | --- | --- | --- | --- | --- | --- | --- | --- | --- | --- | --- | --- |
| A | - | - | - | - | - | - | - | 7 | 8 | 2 | 6 | 1 | 2 |  |  |  |  | 1 | 27 |
| T | - | - | - | - | - | - | - | 16 | 10 | 3 | 5 | 1 |  |  | 1 |  |  |  | 36 |
| AT | - | - | 6 | 1 |  |  |  |  |  |  |  |  |  |  |  |  |  |  | 7 |
| CT | - | - | 1 |  |  |  |  |  |  |  |  |  |  |  |  |  |  |  | 1 |
| TA | - | - | 3 | 1 |  |  |  |  |  |  |  |  |  |  |  |  |  |  | 4 |
| AAT | - | 1 |  |  |  |  |  |  |  |  |  |  |  |  |  |  |  |  | 1 |
| ATT | - | 1 |  |  |  |  |  |  |  |  |  |  |  |  |  |  |  |  | 1 |
| AAAT | 1 |  |  |  |  |  |  |  |  |  |  |  |  |  |  |  |  |  | 1 |
| AATA | 1 |  |  |  |  |  |  |  |  |  |  |  |  |  |  |  |  |  | 1 |
| AATT | 1 |  |  |  |  |  |  |  |  |  |  |  |  |  |  |  |  |  | 1 |
| AGGT | 1 |  |  |  |  |  |  |  |  |  |  |  |  |  |  |  |  |  | 1 |
| ATAA | 1 | 1 |  |  |  |  |  |  |  |  |  |  |  |  |  |  |  |  | 2 |
| CTAC | 1 |  |  |  |  |  |  |  |  |  |  |  |  |  |  |  |  |  | 1 |
| GGAA | 1 |  |  |  |  |  |  |  |  |  |  |  |  |  |  |  |  |  | 1 |
| TAAT | 1 |  |  |  |  |  |  |  |  |  |  |  |  |  |  |  |  |  | 1 |
| TATT | 1 |  |  |  |  |  |  |  |  |  |  |  |  |  |  |  |  |  | 1 |
| TCTT | 1 |  |  |  |  |  |  |  |  |  |  |  |  |  |  |  |  |  | 1 |
| TGAA | 1 |  |  |  |  |  |  |  |  |  |  |  |  |  |  |  |  |  | 1 |
| TTAA | 1 |  |  |  |  |  |  |  |  |  |  |  |  |  |  |  |  |  | 1 |
| TTTC | 1 |  |  |  |  |  |  |  |  |  |  |  |  |  |  |  |  |  | 1 |
| AATAA | 1 |  |  |  |  |  |  |  |  |  |  |  |  |  |  |  |  |  | 1 |
| TTTTC | 1 |  |  |  |  |  |  |  |  |  |  |  |  |  |  |  |  |  | 1 |

**Supplementary table 7:** Frequency of complementary simple sequence repeats in *C. sabulosa.*

| **Repeats** | **3** | **4** | **5** | **6** | **7** | **8** | **9** | **10** | **11** | **12** | **13** | **14** | **15** | **16** | **17** | **18** | **19** | **20** | **Total** |
| --- | --- | --- | --- | --- | --- | --- | --- | --- | --- | --- | --- | --- | --- | --- | --- | --- | --- | --- | --- |
| A/T | - | - | - | - | - | - | - | 23 | 18 | 5 | 11 | 2 | 2 |  | 1 |  |  | 1 | 63 |
| AG/CT | - | - | 1 |  |  |  |  |  |  |  |  |  |  |  |  |  |  |  | 1 |
| AT/AT | - | - | 9 | 2 |  |  |  |  |  |  |  |  |  |  |  |  |  |  | 11 |
| AAT/ATT | - | 2 |  |  |  |  |  |  |  |  |  |  |  |  |  |  |  |  | 2 |
| AAAG/CTTT | 2 |  |  |  |  |  |  |  |  |  |  |  |  |  |  |  |  |  | 2 |
| AAAT/ATTT | 4 | 1 |  |  |  |  |  |  |  |  |  |  |  |  |  |  |  |  | 5 |
| AAGG/CCTT | 1 |  |  |  |  |  |  |  |  |  |  |  |  |  |  |  |  |  | 1 |
| AATG/ATTC | 1 |  |  |  |  |  |  |  |  |  |  |  |  |  |  |  |  |  | 1 |
| AATT/AATT | 3 |  |  |  |  |  |  |  |  |  |  |  |  |  |  |  |  |  | 3 |
| ACCT/AGGT | 2 |  |  |  |  |  |  |  |  |  |  |  |  |  |  |  |  |  | 2 |
| AAAAG/CTTTT | 1 |  |  |  |  |  |  |  |  |  |  |  |  |  |  |  |  |  | 1 |
| AAAAT/ATTTT | 1 |  |  |  |  |  |  |  |  |  |  |  |  |  |  |  |  |  | 1 |

**Supplementary table 8**: Oligonucleotide repeats analysis in *C. sabulosa.*

| **Size** | **Position 1** | **Type** | **Position 2** | **Location** | **Region** | **IGS/CDS/Intron** |
| --- | --- | --- | --- | --- | --- | --- |
|  |  |  |  |  |  |  |
| 28 | 207 | C | 31663 | *trnH-GUG-psbA/trnE-UUC-trnT-GGU* | LSC | IGS |
| 27 | 208 | R | 71280 | *trnH-GUG-psbA/rpl33-rps18* | LSC | IGS |
| 27 | 291 | P | 4472 | *trnH-GUG-psbA/trnK-UUU-rps16* | LSC | IGS |
| 27 | 4444 | P | 8242 | *trnK-UUU-rps16/trnS-GCU-trnS-CGA* | LSC | IGS |
| 28 | 4446 | R | 4446 | *trnK-UUU-rps16* | LSC | IGS |
| 28 | 4452 | R | 84327 | *trnK-UUU-rps16/rps8-rpl14* | LSC | IGS |
| 28 | 4458 | P | 8247 | *trnK-UUU-rps16/trnS-GCU-trnS-CGA* | LSC | IGS |
| 27 | 4460 | C | 8237 | *trnK-UUU-rps16/trnS-GCU-trnS-CGA* | LSC | IGS |
| 24 | 4462 | P | 8247 | *trnK-UUU-rps16/trnS-GCU-trnS-CGA* | LSC | IGS |
| 27 | 6151 | F | 8234 | *rps16-trnQ-UUG/trnS-GCU-trnS-CGA* | LSC | IGS |
| 24 | 6154 | C | 47814 | *rps16-trnQ-UUG/rps4-trnT-UGU* | LSC | IGS |
| 29 | 7641 | F | 36258 | *psbI-trnS-GCU/psbC-trnS-UGA* | LSC | IGS |
| 30 | 7643 | P | 46824 | *psbI-trnS-GCU/trnS-GGA* | LSC | trn/IGS |
| 25 | 7645 | F | 36261 | *trnS-GCU/psbC-trnS-UGA* | LSC | trn/IGS |
| 21 | 7649 | F | 36265 | *trnS-GCU/trnS-UGA* | LSC | trn |
| 27 | 7870 | P | 52191 | *trnS-GCU-trnS-CGA/ndhK* | LSC | IGS/CDS |
| 29 | 7871 | R | 7871 | *trnS-GCU-trnS-CGA* | LSC | IGS |
| 24 | 8237 | C | 47814 | *trnS-GCU-trnS-CGA/rps4-trnT-UGU* | LSC | IGS |
| 26 | 9472 | F | 37292 | *trnS-CGA/trnG-UCC* | LSC | trn |
| 31 | 12216 | P | 12216 | *atpF* | LSC | Intron |
| 27 | 28459 | R | 28459 | *petN-psbM* | LSC | IGS |
| 29 | 29983 | P | 29983 | *psbM-trnD-GUC* | LSC | IGS |
| 22 | 29986 | P | 29986 | *psbM-trnD-GUC* | LSC | IGS |
| 28 | 31639 | P | 71845 | *trnE-UUC-trnT-GGU/rps18* | LSC | IGS/CDS |
| 20 | 31651 | P | 71301 | *trnE-UUC-trnT-GGU/rpl33-rps18* | LSC | IGS |
| 26 | 31654 | F | 48517 | *trnE-UUC-trnT-GGU/trnT-UGU-trnL-UAA* | LSC | IGS |
| 32 | 31660 | P | 71278 | *trnE-UUC-trnT-GGU/rpl33-rps18* | LSC | IGS |
| 28 | 31661 | F | 71166 | *trnE-UUC-trnT-GGU/rpl33-rps18* | LSC | IGS |
| 32 | 31664 | P | 71273 | *trnE-UUC-trnT-GGU/rpl33-rps18* | LSC | IGS |
| 27 | 31864 | C | 43266 | *trnE-UUC-trnT-GGU/psaA-ycf3* | LSC | IGS |
| 30 | 31880 | P | 112395 | *trnE-UUC-trnT-GGU/trnN-GUU-ndhF* | LSC | IGS |
| 25 | 31885 | F | 112394 | *trnE-UUC-trnT-GGU/trnN-GUU-ndhF* | LSC | IGS |
| 24 | 31886 | P | 31886 | *trnE-UUC-trnT-GGU* | LSC | IGS |
| 28 | 31888 | C | 71255 | *trnE-UUC-trnT-GGU/rpl33-rps18* | LSC | IGS |
| 24 | 31892 | C | 71259 | *trnE-UUC-trnT-GGU/rpl33-rps18* | LSC | IGS |
| 22 | 33419 | F | 33441 | *trnT-GGU-psbD* | LSC | IGS |
| 25 | 36261 | P | 46827 | *trnS-UGA/trnS-GGA* | LSC | trn |
| 24 | 36333 | P | 46762 | *trnS-UGA/ycf3-trnS-GGA* | LSC | trn/IGS |
| 30 | 39507 | F | 41731 | *psaB/psaA* | LSC | CDS |
| 27 | 43385 | P | 43385 | *psaA-ycf3* | LSC | IGS |
| 27 | 44923 | F | 101854 | *ycf3/rps12-trnV-GAC* | LSC/IR | Intron/IGS |
| 27 | 44923 | F | 122895 | *ycf3/ndhA* | LSC/SSC | Intron |
| 28 | 49459 | F | 97554 | *trnL-UAA/trnL-CAA-ndhB* | LSC/IR | Intron/IGS |
| 30 | 49462 | F | 97559 | *trnL-UAA/trnL-CAA-ndhB* | LSC/IR | Intron/IGS |
| 20 | 52380 | R | 52380 | *ndhC* | LSC | CDS |
| 27 | 53298 | F | 53320 | *ndhC-trnC-ACA* | LSC | IGS |
| 20 | 54623 | P | 54623 | *trnC-ACA-trnM-CAU* | LSC | IGS |
| 20 | 54984 | P | 54984 | *trnM-CAU-atpE* | LSC | IGS |
| 27 | 59462 | F | 59485 | *rbcL-accD* | LSC | IGS |
| 22 | 67822 | R | 67822 | *psbE-petL* | LSC | IGS |
| 27 | 70501 | F | 126183 | *psaJ-rpl33/rps15-ycf1* | LSC/SSC | IGS |
| 20 | 70501 | R | 70501 | *psaJ-rpl33* | LSC | IGS |
| 20 | 70504 | P | 85645 | *psaJ-rpl33/rpl16-rps3* | LSC | IGS |
| 31 | 71250 | R | 71260 | *rpl33-rps18* | LSC | IGS |
| 31 | 71260 | R | 71277 | *rpl33-rps18* | LSC | IGS |
| 32 | 71287 | R | 71287 | *rpl33-rps18* | LSC | IGS |
| 29 | 71844 | R | 71844 | *rps18* | LSC | CDS |
| 20 | 73181 | R | 73181 | *rpl20-rps12* | LSC | IGS |
| 44 | 77924 | P | 77924 | *psbT-psbN* | LSC | IGS |
| 25 | 79065 | R | 79065 | *psbH-petB* | LSC | IGS |
| 20 | 80191 | P | 80191 | *petB-petD* | LSC | IGS |
| 30 | 94549 | F | 94609 | *ycf2* | IR | CDS |
| 23 | 94556 | F | 94616 | *ycf2* | IR | CDS |
| 51 | 94577 | F | 94595 | *ycf2* | IR | CDS |
| 33 | 94577 | F | 94613 | *ycf2* | IR | CDS |
| 42 | 101840 | F | 122881 | *rps12-trnV-GAC/ndhA* | IR/SSC | Intron/IGS |
| 33 | 112367 | R | 112373 | *trnN-GUU-ndhF* | SSC | IGS |
| 45 | 112453 | R | 112464 | *trnN-GUU-ndhF* | SSC | IGS |
| 31 | 112453 | F | 112472 | *trnN-GUU-ndhF* | SSC | IGS |
| 28 | 112456 | F | 112476 | *trnN-GUU-ndhF* | SSC | IGS |
| 41 | 112457 | R | 112464 | *trnN-GUU-ndhF* | SSC | IGS |
| 25 | 112459 | F | 112478 | *trnN-GUU-ndhF* | SSC | IGS |
| 20 | 112464 | F | 112483 | *trnN-GUU-ndhF* | SSC | IGS |
| 32 | 112471 | R | 112478 | *trnN-GUU-ndhF* | SSC | IGS |
| 20 | 114062 | P | 114062 | *ndhF* | SSC | CDS |
| 24 | 117828 | F | 117851 | *ccsA-ndhD* | SSC | IGS |
| 44 | 117859 | P | 117859 | *ccsA-ndhD* | SSC | IGS |
| 24 | 119441 | P | 119467 | *ndhD* | LSC | CDS |

**Supplementary table 9:** Non-synonymous (Ka) and synonymous rate (Ks) of substitution, Ka/Ks value in *C. sabulosa* by making pairwise alignment with four species of Euphorbiaceae (*Ricinus communis*, *Jatropha curcas*, *Euphorbia helioscopia,* and *Manihot esculenta*) and four Phyllanthaceae species (*Antidesma bunius*, *Breynia fruticosa*, *Glochidion chodoense*, *and Phyllanthus urinaria*).

| **Sr.No** | **GENES** | **Species which Pairwise aligned with *Chrozophora sabulosa*** | **Ka** | **Ks** | **Ka/Ks** |
| --- | --- | --- | --- | --- | --- |
| 1 | ***psbA*** | *Ricinus communis* | 0.0012 | 0.2116 | 0.005671078 |
|  |  | *Jatropha curcas* | 0 | 0.1752 | 0 |
|  |  | *Euphorbia helioscopia* | 0.0012 | 0.2166 | 0.005540166 |
|  |  | *Manihot esculenta* | 0.0012 | 0.1647 | 0.007285974 |
|  |  | *Antidesma bunius* | 0.0025 | 0.1903 | 0.013137152 |
|  |  | *Breynia fruticosa* | 0.0025 | 0.1905 | 0.01312336 |
|  |  | *Glochidion chodoense* | 0.0025 | 0.2121 | 0.011786893 |
|  |  | *Phyllanthus urinaria* | 0.0025 | 0.2012 | 0.012425447 |
| 2 | ***matK*** | *Ricinus communis* | 0.1607 | 0.3871 | 0.415138207 |
|  |  | *Jatropha curcas* | 0.1725 | 0.4168 | 0.413867562 |
|  |  | *Euphorbia helioscopia* | 0.1811 | 0.4023 | 0.450161571 |
|  |  | *Manihot esculenta* | 0.1526 | 0.3698 | 0.412655489 |
|  |  | *Antidesma bunius* | 0.1869 | 0.4183 | 0.446808511 |
|  |  | *Breynia fruticosa* | 0.1793 | 0.405 | 0.442716049 |
|  |  | *Glochidion chodoense* | 0.182 | 0.4284 | 0.424836601 |
|  |  | *Phyllanthus urinaria* | 0.1857 | 0.3956 | 0.469413549 |
| 3 | ***rps16*** | *Ricinus communis* | 0.1002 | 0.3501 | 0.286203942 |
|  |  | *Jatropha curcas* | 0 | 0 | 0 |
|  |  | *Euphorbia helioscopia* | 0 | 0 | 0 |
|  |  | *Manihot esculenta* | 0.0714 | 0.263 | 0.27148289 |
|  |  | *Antidesma bunius* | 0.0524 | 0.3262 | 0.160637646 |
|  |  | *Breynia fruticosa* | 0.0844 | 0.3326 | 0.253758268 |
|  |  | *Glochidion chodoense* | 0.0765 | 0.5221 | 0.146523654 |
|  |  | *Phyllanthus urinaria* | 0.0846 | 0.3295 | 0.256752656 |
| 4 | ***psbK*** | *Ricinus communis* | 0.0452 | 0.4236 | 0.106704438 |
|  |  | *Jatropha curcas* | 0.0374 | 0.3852 | 0.09709242 |
|  |  | *Euphorbia helioscopia* | 0.0531 | 0.2995 | 0.177295492 |
|  |  | *Manihot esculenta* | 0.0763 | 0.3938 | 0.193753174 |
|  |  | *Antidesma bunius* | 0.0617 | 0.3155 | 0.195562599 |
|  |  | *Breynia fruticosa* | 0.0452 | 0.4213 | 0.107286969 |
|  |  | *Glochidion chodoense* | 0.0461 | 0.4308 | 0.107010214 |
|  |  | *Phyllanthus urinaria* | 0.0451 | 0.4715 | 0.095652174 |
| 5 | ***atpA*** | *Ricinus communis* | 0.0088 | 0.4017 | 0.021906896 |
|  |  | *Jatropha curcas* | 0.0172 | 0.3802 | 0.045239348 |
|  |  | *Euphorbia helioscopia* | 0.015 | 0.4451 | 0.033700292 |
|  |  | *Manihot esculenta* | 0.0123 | 0.3398 | 0.036197763 |
|  |  | *Antidesma bunius* | 0.0158 | 0.3836 | 0.041188738 |
|  |  | *Breynia fruticosa* | 0.0167 | 0.392 | 0.042602041 |
|  |  | *Glochidion chodoense* | 0.0176 | 0.3745 | 0.046995995 |
|  |  | *Phyllanthus urinaria* | 0.0167 | 0.3921 | 0.042591176 |
| 6 | ***psbI*** | *Ricinus communis* | 0 | 0.3256 | 0 |
|  |  | *Jatropha curcas* | 0 | 0.2685 | 0 |
|  |  | *Euphorbia helioscopia* | 0 | 0.6106 | 0 |
|  |  | *Manihot esculenta* | 0 | 0.2189 | 0 |
|  |  | *Antidesma bunius* | 0 | 0.3282 | 0 |
|  |  | *Breynia fruticosa* | 0 | 0.3895 | 0 |
|  |  | *Glochidion chodoense* | 0 | 0.3895 | 0 |
|  |  | *Phyllanthus urinaria* | 0 | 0.3895 | 0 |
| 7 | ***atpH*** | *Ricinus communis* | 0 | 0.4141 | 0 |
|  |  | *Jatropha curcas* | 0 | 0.2928 | 0 |
|  |  | *Euphorbia helioscopia* | 0 | 0.3175 | 0 |
|  |  | *Manihot esculenta* | 0 | 0.3379 | 0 |
|  |  | *Antidesma bunius* | 0 | 0.2708 | 0 |
|  |  | *Breynia fruticosa* | 0 | 0.5587 | 0 |
|  |  | *Glochidion chodoense* | 0 | 0.5275 | 0 |
|  |  | *Phyllanthus urinaria* | 0.0057 | 0.4993 | 0.011415982 |
| 8 | ***rpoC2*** | *Ricinus communis* | 0.0733 | 0.3316 | 0.221049457 |
|  |  | *Jatropha curcas* | 0.0739 | 0.3451 | 0.214140829 |
|  |  | *Euphorbia helioscopia* | 0.0838 | 0.3537 | 0.236923947 |
|  |  | *Manihot esculenta* | 0.0697 | 0.321 | 0.217133956 |
|  |  | *Antidesma bunius* | 0.077 | 0.3526 | 0.218377765 |
|  |  | *Breynia fruticosa* | 0.0794 | 0.3669 | 0.216407741 |
|  |  | *Glochidion chodoense* | 0.0805 | 0.363 | 0.221763085 |
|  |  | *Phyllanthus urinaria* | 0.0823 | 0.3857 | 0.213378273 |
| 9 | ***petN*** | *Ricinus communis* | 0 | 0.2218 | 0 |
|  |  | *Jatropha curcas* | 0 | 0.1027 | 0 |
|  |  | *Euphorbia helioscopia* | 0 | 0.1027 | 0 |
|  |  | *Manihot esculenta* | 0 | 0.1027 | 0 |
|  |  | *Antidesma bunius* | 0 | 0.2218 | 0 |
|  |  | *Breynia fruticosa* | 0 | 0.1585 | 0 |
|  |  | *Glochidion chodoense* | 0 | 0.2208 | 0 |
|  |  | *Phyllanthus urinaria* | 0 | 0.1585 | 0 |
| 10 | ***psbD*** | *Ricinus communis* | 0.0037 | 0.2553 | 0.014492754 |
|  |  | *Jatropha curcas* | 0.0025 | 0.2613 | 0.009567547 |
|  |  | *Euphorbia helioscopia* | 0.0037 | 0.291 | 0.012714777 |
|  |  | *Manihot esculenta* | 0.0037 | 0.2506 | 0.014764565 |
|  |  | *Antidesma bunius* | 0.005 | 0.2508 | 0.019936204 |
|  |  | *Breynia fruticosa* | 0.0037 | 0.272 | 0.013602941 |
|  |  | *Glochidion chodoense* | 0.0037 | 0.2722 | 0.013592946 |
|  |  | *Phyllanthus urinaria* | 0.0037 | 0.2778 | 0.013318934 |
| 11 | ***psbC*** | *Ricinus communis* | 0.0052 | 0.3048 | 0.017060367 |
|  |  | *Jatropha curcas* | 0.0052 | 0.2841 | 0.018303414 |
|  |  | *Euphorbia helioscopia* | 0.008 | 0.3179 | 0.025165146 |
|  |  | *Manihot esculenta* | 0.0053 | 0.3017 | 0.01756712 |
|  |  | *Antidesma bunius* | 0.0066 | 0.2777 | 0.023766655 |
|  |  | *Breynia fruticosa* | 0.0075 | 0.3196 | 0.023466834 |
|  |  | *Glochidion chodoense* | 0.0085 | 0.3112 | 0.027313625 |
|  |  | *Phyllanthus urinaria* | 0.0066 | 0.32 | 0.020625 |
| 12 | ***ycf3*** | *Ricinus communis* | 0.013 | 0.2367 | 0.054921842 |
|  |  | *Jatropha curcas* | 0.0183 | 0.2507 | 0.072995612 |
|  |  | *Euphorbia helioscopia* | 0.0158 | 0.3028 | 0.052179657 |
|  |  | *Manihot esculenta* | 0.013 | 0.2633 | 0.049373338 |
|  |  | *Antidesma bunius* | 0.0183 | 0.3028 | 0.060435931 |
|  |  | *Breynia fruticosa* | 0.013 | 0.2872 | 0.045264624 |
|  |  | *Glochidion chodoense* | 0.013 | 0.2843 | 0.045726345 |
|  |  | *Phyllanthus urinaria* | 0.0157 | 0.2753 | 0.057028696 |
| 13 | ***rps4*** | *Ricinus communis* | 0.0462 | 0.2467 | 0.18727199 |
|  |  | *Jatropha curcas* | 0.0498 | 0.2298 | 0.216710183 |
|  |  | *Euphorbia helioscopia* | 0.0473 | 0.2814 | 0.168088131 |
|  |  | *Manihot esculenta* | 0.0404 | 0.178 | 0.226966292 |
|  |  | *Antidesma bunius* | 0.0495 | 0.2742 | 0.180525164 |
|  |  | *Breynia fruticosa* | 0.0495 | 0.3144 | 0.157442748 |
|  |  | *Glochidion chodoense* | 0.0495 | 0.3043 | 0.162668419 |
|  |  | *Phyllanthus urinaria* | 0.0483 | 0.2899 | 0.166609176 |
| 14 | ***ndhK*** | *Ricinus communis* | 0.1599 | 0.1425 | 1.122105263 |
|  |  | *Jatropha curcas* | 0.0361 | 0.3495 | 0.103290415 |
|  |  | *Euphorbia helioscopia* | 0.0467 | 0.425 | 0.109882353 |
|  |  | *Manihot esculenta* | 0.0341 | 0.3312 | 0.102958937 |
|  |  | *Antidesma bunius* | 0.0484 | 0.3947 | 0.122624778 |
|  |  | *Breynia fruticosa* | 0.0586 | 0.3698 | 0.158464035 |
|  |  | *Glochidion chodoense* | 0.0487 | 0.3758 | 0.129590208 |
|  |  | *Phyllanthus urinaria* | 0.0361 | 0.3409 | 0.105896157 |
| 15 | ***atpE*** | *Ricinus communis* | 0.0438 | 0.3467 | 0.126334006 |
|  |  | *Jatropha curcas* | 0.0302 | 0.2784 | 0.108477011 |
|  |  | *Euphorbia helioscopia* | 0.0342 | 0.356 | 0.096067416 |
|  |  | *Manihot esculenta* | 0.037 | 0.2647 | 0.139780884 |
|  |  | *Antidesma bunius* | 0.0336 | 0.3108 | 0.108108108 |
|  |  | *Breynia fruticosa* | 0.044 | 0.3593 | 0.12246034 |
|  |  | *Glochidion chodoense* | 0.0445 | 0.3396 | 0.131036514 |
|  |  | *Phyllanthus urinaria* | 0.0492 | 0.3691 | 0.133297209 |
| 16 | ***rbcL*** | *Ricinus communis* | 0.0343 | 0.2963 | 0.115761053 |
|  |  | *Jatropha curcas* | 0.0356 | 0.3079 | 0.115621955 |
|  |  | *Euphorbia helioscopia* | 0.0411 | 0.3406 | 0.120669407 |
|  |  | *Manihot esculenta* | 0.0381 | 0.3066 | 0.124266145 |
|  |  | *Antidesma bunius* | 0.0416 | 0.3515 | 0.118349929 |
|  |  | *Breynia fruticosa* | 0.0445 | 0.3338 | 0.133313361 |
|  |  | *Glochidion chodoense* | 0.0492 | 0.3321 | 0.148148148 |
|  |  | *Phyllanthus urinaria* | 0.041 | 0.3729 | 0.109949048 |
| 17 | ***accD*** | *Ricinus communis* | 0.1048 | 0.3261 | 0.321373812 |
|  |  | *Jatropha curcas* | 0.0953 | 0.2925 | 0.325811966 |
|  |  | *Euphorbia helioscopia* | 0.1149 | 0.3903 | 0.294388932 |
|  |  | *Manihot esculenta* | 0.112 | 0.3485 | 0.321377331 |
|  |  | *Antidesma bunius* | 0.1205 | 0.3561 | 0.338388093 |
|  |  | *Breynia fruticosa* | 0.1122 | 0.4134 | 0.271407837 |
|  |  | *Glochidion chodoense* | 0.1101 | 0.409 | 0.269193154 |
|  |  | *Phyllanthus urinaria* | 0.1091 | 0.4252 | 0.256585136 |
| 18 | ***ycf4*** | *Ricinus communis* | 0.0338 | 0.3276 | 0.103174603 |
|  |  | *Jatropha curcas* | 0.0439 | 0.3 | 0.146333333 |
|  |  | *Euphorbia helioscopia* | 0.0676 | 0.3138 | 0.215423837 |
|  |  | *Manihot esculenta* | 0.0388 | 0.2786 | 0.139267767 |
|  |  | *Antidesma bunius* | 0.0461 | 0.318 | 0.144968553 |
|  |  | *Breynia fruticosa* | 0.0646 | 0.3858 | 0.167444272 |
|  |  | *Glochidion chodoense* | 0.0569 | 0.3723 | 0.152833736 |
|  |  | *Phyllanthus urinaria* | 0.0673 | 0.3701 | 0.181842745 |
| 19 | ***cemA*** | *Ricinus communis* | 0.0632 | 0.3831 | 0.164969982 |
|  |  | *Jatropha curcas* | 0.0636 | 0.3378 | 0.188277087 |
|  |  | *Euphorbia helioscopia* | 0.0885 | 0.322 | 0.27484472 |
|  |  | *Manihot esculenta* | 0.0711 | 0.2848 | 0.249648876 |
|  |  | *Antidesma bunius* | 0.1389 | 0.0727 | 1.910591472 |
|  |  | *Breynia fruticosa* | 0.0801 | 0.3847 | 0.208214193 |
|  |  | *Glochidion chodoense* | 0.0688 | 0.3769 | 0.182541788 |
|  |  | *Phyllanthus urinaria* | 0.0706 | 0.361 | 0.195567867 |
| 20 | ***psbF*** | *Ricinus communis* | 0.0236 | 0.0332 | 0.710843373 |
|  |  | *Jatropha curcas* | 0 | 0.0678 | 0 |
|  |  | *Euphorbia helioscopia* | 0.0117 | 0.0678 | 0.172566372 |
|  |  | *Manihot esculenta* | 0 | 0.0328 | 0 |
|  |  | *Antidesma bunius* | 0.0117 | 0.1042 | 0.112284069 |
|  |  | *Breynia fruticosa* | 0 | 0.103 | 0 |
|  |  | *Glochidion chodoense* | 0 | 0.103 | 0 |
|  |  | *Phyllanthus urinaria* | 0 | 0.103 | 0 |
| 21 | ***psbE*** | *Ricinus communis* | 0.0105 | 0.1956 | 0.053680982 |
|  |  | *Jatropha curcas* | 0.0105 | 0.1314 | 0.079908676 |
|  |  | *Euphorbia helioscopia* | 0.0105 | 0.2417 | 0.043442284 |
|  |  | *Manihot esculenta* | 0.0105 | 0.2183 | 0.048098946 |
|  |  | *Antidesma bunius* | 0.0105 | 0.2908 | 0.03610729 |
|  |  | *Breynia fruticosa* | 0.0106 | 0.1517 | 0.069874753 |
|  |  | *Glochidion chodoense* | 0.0106 | 0.1108 | 0.09566787 |
|  |  | *Phyllanthus urinaria* | 0.0106 | 0.1517 | 0.069874753 |
| 22 | ***petL*** | *Ricinus communis* | 0.0614 | 0.2869 | 0.214011851 |
|  |  | *Jatropha curcas* | 0.0455 | 0.349 | 0.130372493 |
|  |  | *Euphorbia helioscopia* | 0.0451 | 0.3597 | 0.125382263 |
|  |  | *Manihot esculenta* | 0.0456 | 0.2869 | 0.158940397 |
|  |  | *Antidesma bunius* | 0.0456 | 0.4135 | 0.110278114 |
|  |  | *Breynia fruticosa* | 0.0774 | 0.3505 | 0.220827389 |
|  |  | *Glochidion chodoense* | 0.0776 | 0.4135 | 0.187666264 |
|  |  | *Phyllanthus urinaria* | 0.0776 | 0.4135 | 0.187666264 |
| 23 | ***petG*** | *Ricinus communis* | 0.0121 | 0.159 | 0.076100629 |
|  |  | *Jatropha curcas* | 0.0122 | 0.1134 | 0.107583774 |
|  |  | *Euphorbia helioscopia* | 0.0122 | 0.2506 | 0.04868316 |
|  |  | *Manihot esculenta* | 0 | 0.1149 | 0 |
|  |  | *Antidesma bunius* | 0.0121 | 0.2082 | 0.058117195 |
|  |  | *Breynia fruticosa* | 0.0122 | 0.1559 | 0.078255292 |
|  |  | *Glochidion chodoense* | 0.0122 | 0.1559 | 0.078255292 |
|  |  | *Phyllanthus urinaria* | 0.0245 | 0.2033 | 0.120511559 |
| 24 | ***rps18*** | *Ricinus communis* | 0.0618 | 0.2421 | 0.255266419 |
|  |  | *Jatropha curcas* | 0.0895 | 0.2105 | 0.425178147 |
|  |  | *Euphorbia helioscopia* | 0.0593 | 0.2762 | 0.214699493 |
|  |  | *Manihot esculenta* | 0.0669 | 0.2187 | 0.305898491 |
|  |  | *Antidesma bunius* | 0.0625 | 0.2297 | 0.272094036 |
|  |  | *Breynia fruticosa* | 0.0816 | 0.2442 | 0.334152334 |
|  |  | *Glochidion chodoense* | 0.0818 | 0.2624 | 0.311737805 |
|  |  | *Phyllanthus urinaria* | 0.0816 | 0.2442 | 0.334152334 |
| 25 | ***clpP*** | *Ricinus communis* | 0.0754 | 0.2543 | 0.296500197 |
|  |  | *Jatropha curcas* | 0.0816 | 0.2201 | 0.370740572 |
|  |  | *Euphorbia helioscopia* | 0.0842 | 0.2097 | 0.40152599 |
|  |  | *Manihot esculenta* | 0.0768 | 0.2479 | 0.30980234 |
|  |  | *Antidesma bunius* | 0.0827 | 0.2664 | 0.310435435 |
|  |  | *Breynia fruticosa* | 0.0854 | 0.2153 | 0.396655829 |
|  |  | *Glochidion chodoense* | 0.0968 | 0.2298 | 0.421235857 |
|  |  | *Phyllanthus urinaria* | 0.0778 | 0.2158 | 0.360518999 |
| 26 | ***psbT*** | *Ricinus communis* | 0 | 0.4408 | 0 |
|  |  | *Jatropha curcas* | 0 | 0.4347 | 0 |
|  |  | *Euphorbia helioscopia* | 0 | 0.5122 | 0 |
|  |  | *Manihot esculenta* | 0 | 0.5122 | 0 |
|  |  | *Antidesma bunius* | 0.0265 | 0.1793 | 0.147796988 |
|  |  | *Breynia fruticosa* | 0.0263 | 0.2928 | 0.089822404 |
|  |  | *Glochidion chodoense* | 0.0263 | 0.2928 | 0.089822404 |
|  |  | *Phyllanthus urinaria* | 0 | 0.1834 | 0 |
| 27 | ***psbN*** | *Ricinus communis* | 0 | 0.1762 | 0 |
|  |  | *Jatropha curcas* | 0.0104 | 0.2145 | 0.048484848 |
|  |  | *Euphorbia helioscopia* | 0.0104 | 0.2145 | 0.048484848 |
|  |  | *Manihot esculenta* | 0.0104 | 0.1742 | 0.059701493 |
|  |  | *Antidesma bunius* | 0 | 0.3549 | 0 |
|  |  | *Breynia fruticosa* | 0.0104 | 0.4029 | 0.025812857 |
|  |  | *Glochidion chodoense* | 0.0104 | 0.4029 | 0.025812857 |
|  |  | *Phyllanthus urinaria* | 0.0104 | 0.3513 | 0.029604327 |
| 28 | ***psbH*** | *Ricinus communis* | 0.0573 | 0.2286 | 0.250656168 |
|  |  | *Jatropha curcas* | 0.0507 | 0.2294 | 0.221011334 |
|  |  | *Euphorbia helioscopia* | 0.064 | 0.2524 | 0.253565769 |
|  |  | *Manihot esculenta* | 0.0441 | 0.2808 | 0.157051282 |
|  |  | *Antidesma bunius* | 0.0638 | 0.1167 | 0.546700943 |
|  |  | *Breynia fruticosa* | 0.0635 | 0.1622 | 0.391491985 |
|  |  | *Glochidion chodoense* | 0.0669 | 0.1737 | 0.385146805 |
|  |  | *Phyllanthus urinaria* | 0.0635 | 0.1399 | 0.45389564 |
| 29 | ***petD*** | *Ricinus communis* | 0.0854 | 0.0094 | 9.09 |
|  |  | *Jatropha curcas* | 0.0111 | 0.3771 | 0.03 |
|  |  | *Euphorbia helioscopia* | 0.1076 | 0.019 | 5.66 |
|  |  | *Manihot esculenta* | 0.0111 | 0.2975 | 0.04 |
|  |  | *Antidesma bunius* | 0.0195 | 0.3465 | 0.06 |
|  |  | *Breynia fruticosa* | 0.0223 | 0.3744 | 0.06 |
|  |  | *Glochidion chodoense* | 0.0993 | 0.0189 | 5.25 |
|  |  | *Phyllanthus urinaria* | 0.0195 | 0.3465 | 0.06 |
| 30 | ***rpl36*** | *Ricinus communis* | 0.0242 | 0.374 | 0.064705882 |
|  |  | *Jatropha curcas* | 0.0242 | 0.3157 | 0.076655052 |
|  |  | *Euphorbia helioscopia* | 0.0241 | 0.5175 | 0.046570048 |
|  |  | *Manihot esculenta* | 0.0242 | 0.4372 | 0.055352242 |
|  |  | *Antidesma bunius* | 0.012 | 0.3816 | 0.031446541 |
|  |  | *Breynia fruticosa* | 0.0121 | 0.3681 | 0.032871502 |
|  |  | *Glochidion chodoense* | 0.0121 | 0.4283 | 0.028251226 |
|  |  | *Phyllanthus urinaria* | 0.0243 | 0.43 | 0.056511628 |
| 31 | ***rpl14*** | *Ricinus communis* | 0.03 | 0.3951 | 0.075930144 |
|  |  | *Jatropha curcas* | 0.0339 | 0.3566 | 0.095064498 |
|  |  | *Euphorbia helioscopia* | 0.0417 | 0.3916 | 0.10648621 |
|  |  | *Manihot esculenta* | 0.03 | 0.4151 | 0.072271742 |
|  |  | *Antidesma bunius* | 0.0338 | 0.3765 | 0.089774236 |
|  |  | *Breynia fruticosa* | 0.0262 | 0.4714 | 0.055579126 |
|  |  | *Glochidion chodoense* | 0.03 | 0.4757 | 0.063064957 |
|  |  | *Phyllanthus urinaria* | 0.0301 | 0.5377 | 0.055979171 |
| 32 | ***rpl16*** | *Ricinus communis* | 0.023 | 0.3363 | 0.068391317 |
|  |  | *Jatropha curcas* | 0.0774 | 0.3856 | 0.200726141 |
|  |  | *Euphorbia helioscopia* | 0.0428 | 0.406 | 0.105418719 |
|  |  | *Manihot esculenta* | 0.0272 | 0.3714 | 0.073236403 |
|  |  | *Antidesma bunius* | 0.0409 | 0.3994 | 0.102403605 |
|  |  | *Breynia fruticosa* | 0.0674 | 0.4761 | 0.141566898 |
|  |  | *Glochidion chodoense* | 0.0765 | 0.5221 | 0.146523654 |
|  |  | *Phyllanthus urinaria* | 0.0638 | 0.5142 | 0.124076235 |
| 33 | ***rpl23*** | *Ricinus communis* | 0.0236 | 0.0485 | 0.486597938 |
|  |  | *Jatropha curcas* | 0.0236 | 0.0485 | 0.486597938 |
|  |  | *Euphorbia helioscopia* | 0.0236 | 0.0485 | 0.486597938 |
|  |  | *Manihot esculenta* | 0.0236 | 0.032 | 0.7375 |
|  |  | *Antidesma bunius* | 0.0189 | 0.0482 | 0.392116183 |
|  |  | *Breynia fruticosa* | 0.0481 | 0.0481 | 1 |
|  |  | *Glochidion chodoense* | 0.0531 | 0.0481 | 1.103950104 |
|  |  | *Phyllanthus urinaria* | 0.0481 | 0.0481 | 1 |
| 34 | ***rps7*** | *Ricinus communis* | 0.0144 | 0.0177 | 0.813559322 |
|  |  | *Jatropha curcas* | 0.0086 | 0.036 | 0.238888889 |
|  |  | *Euphorbia helioscopia* | 0.0086 | 0.0178 | 0.483146067 |
|  |  | *Manihot esculenta* | 0.0086 | 0.0178 | 0.483146067 |
|  |  | *Antidesma bunius* | 0.0086 | 0.0267 | 0.322097378 |
|  |  | *Breynia fruticosa* | 0.0086 | 0.0267 | 0.322097378 |
|  |  | *Glochidion chodoense* | 0.0115 | 0.0177 | 0.649717514 |
|  |  | *Phyllanthus urinaria* | 0.0086 | 0.0177 | 0.485875706 |
| 35 | ***ndhF*** | *Ricinus communis* | 0.0993 | 0.486 | 0.204320988 |
|  |  | *Jatropha curcas* | 0.1059 | 0.5052 | 0.209619952 |
|  |  | *Euphorbia helioscopia* | 0.1142 | 0.5068 | 0.225335438 |
|  |  | *Manihot esculenta* | 0.099 | 0.4858 | 0.203787567 |
|  |  | *Antidesma bunius* | 0.11 | 0.4513 | 0.243740306 |
|  |  | *Breynia fruticosa* | 0.1118 | 0.4867 | 0.229710294 |
|  |  | *Glochidion chodoense* | 0.1181 | 0.4904 | 0.240823817 |
|  |  | *Phyllanthus urinaria* | 0.1081 | 0.4974 | 0.217330117 |
| 36 | ***rpl32*** | *Ricinus communis* | 0.1129 | 0.4185 | 0.269772999 |
|  |  | *Jatropha curcas* | 0.0976 | 0.4282 | 0.227930873 |
|  |  | *Euphorbia helioscopia* | 0 | 0 | 0 |
|  |  | *Manihot esculenta* | 0.1161 | 0.4575 | 0.253770492 |
|  |  | *Antidesma bunius* | 0.1176 | 0.4369 | 0.269169146 |
|  |  | *Breynia fruticosa* | 0.1164 | 0.4827 | 0.241143567 |
|  |  | *Glochidion chodoense* | 0.1129 | 0.4493 | 0.251279769 |
|  |  | *Phyllanthus urinaria* | 0.1028 | 0.5025 | 0.204577114 |
| 37 | ***psaC*** | *Ricinus communis* | 0.0054 | 0.5068 | 0.010655091 |
|  |  | *Jatropha curcas* | 0.0054 | 0.4731 | 0.011414077 |
|  |  | *Euphorbia helioscopia* | 0.0054 | 0.4408 | 0.012250454 |
|  |  | *Manihot esculenta* | 0.0054 | 0.3241 | 0.016661524 |
|  |  | *Antidesma bunius* | 0.0054 | 0.3543 | 0.015241321 |
|  |  | *Breynia fruticosa* | 0.0054 | 0.3516 | 0.015358362 |
|  |  | *Glochidion chodoense* | 0.0054 | 0.3241 | 0.016661524 |
|  |  | *Phyllanthus urinaria* | 0.0054 | 0.3241 | 0.016661524 |
| 38 | ***ndhE*** | *Ricinus communis* | 0.0219 | 0.4762 | 0.04598908 |
|  |  | *Jatropha curcas* | 0.0175 | 0.4429 | 0.039512305 |
|  |  | *Euphorbia helioscopia* | 0.0308 | 0.375 | 0.082133333 |
|  |  | *Manihot esculenta* | 0.0263 | 0.3516 | 0.07480091 |
|  |  | *Antidesma bunius* | 0.0177 | 0.4458 | 0.039703903 |
|  |  | *Breynia fruticosa* | 0.0358 | 0.4983 | 0.071844271 |
|  |  | *Glochidion chodoense* | 0.0308 | 0.4472 | 0.068872987 |
|  |  | *Phyllanthus urinaria* | 0.0219 | 0.4208 | 0.052043726 |
| 39 | ***ndhA*** | *Ricinus communis* | 0.0467 | 0.3382 | 0.138083974 |
|  |  | *Jatropha curcas* | 0.0536 | 0.3388 | 0.158205431 |
|  |  | *Euphorbia helioscopia* | 0.0663 | 0.3956 | 0.167593529 |
|  |  | *Manihot esculenta* | 0.0525 | 0.3383 | 0.155187703 |
|  |  | *Antidesma bunius* | 0.0552 | 0.3953 | 0.139640779 |
|  |  | *Breynia fruticosa* | 0.0579 | 0.3456 | 0.167534722 |
|  |  | *Glochidion chodoense* | 0.0593 | 0.3562 | 0.166479506 |
|  |  | *Phyllanthus urinaria* | 0.054 | 0.357 | 0.151260504 |
| 40 | ***ndhH*** | *Ricinus communis* | 0.025 | 0.4287 | 0.058315839 |
|  |  | *Jatropha curcas* | 0.0373 | 0.4452 | 0.08378257 |
|  |  | *Euphorbia helioscopia* | 0.0387 | 0.5323 | 0.072703363 |
|  |  | *Manihot esculenta* | 0.0324 | 0.46 | 0.070434783 |
|  |  | *Antidesma bunius* | 0.0312 | 0.3773 | 0.082692817 |
|  |  | *Breynia fruticosa* | 0.0284 | 0.4673 | 0.060774663 |
|  |  | *Glochidion chodoense* | 0.0307 | 0.481 | 0.063825364 |
|  |  | *Phyllanthus urinaria* | 0.0295 | 0.5025 | 0.058706468 |
| 41 | ***atpF*** | *Ricinus communis* | 0.0419 | 0.2219 | 0.188823795 |
|  |  | *Jatropha curcas* | 0.067 | 0.2296 | 0.291811847 |
|  |  | *Euphorbia helioscopia* | 0.0468 | 0.267 | 0.175280899 |
|  |  | *Manihot esculenta* | 0.0407 | 0.2264 | 0.179770318 |
|  |  | *Antidesma bunius* | 0.0431 | 0.3352 | 0.128579952 |
|  |  | *Breynia fruticosa* | 0.0466 | 0.309 | 0.150809061 |
|  |  | *Glochidion chodoense* | 0.0454 | 0.3148 | 0.144218551 |
|  |  | *Phyllanthus urinaria* | 0.0454 | 0.2664 | 0.17042042 |
| 42 | ***atpI*** | *Ricinus communis* | 0.0265 | 0.3403 | 0.077872465 |
|  |  | *Jatropha curcas* | 0.0328 | 0.3631 | 0.090333242 |
|  |  | *Euphorbia helioscopia* | 0.0319 | 0.3491 | 0.091377829 |
|  |  | *Manihot esculenta* | 0.0301 | 0.306 | 0.098366013 |
|  |  | *Antidesma bunius* | 0.0282 | 0.3487 | 0.08087181 |
|  |  | *Breynia fruticosa* | 0.0285 | 0.3745 | 0.076101469 |
|  |  | *Glochidion chodoense* | 0.0273 | 0.3815 | 0.071559633 |
|  |  | *Phyllanthus urinaria* | 0.031 | 0.3724 | 0.083243824 |
| 43 | ***rps2*** | *Ricinus communis* | 0.0329 | 0.3862 | 0.085189021 |
|  |  | *Jatropha curcas* | 0.0354 | 0.3935 | 0.089961881 |
|  |  | *Euphorbia helioscopia* | 0.0421 | 0.4224 | 0.099668561 |
|  |  | *Manihot esculenta* | 0.0354 | 0.3853 | 0.09187646 |
|  |  | *Antidesma bunius* | 0.0451 | 0.4263 | 0.105794042 |
|  |  | *Breynia fruticosa* | 0.0355 | 0.4351 | 0.081590439 |
|  |  | *Glochidion chodoense* | 0.0471 | 0.4153 | 0.113411991 |
|  |  | *Phyllanthus urinaria* | 0.0374 | 0.4333 | 0.086314332 |
| 44 | ***rpoC1*** | *Ricinus communis* | 0.0312 | 0.2905 | 0.107401033 |
|  |  | *Jatropha curcas* | 0.0323 | 0.2615 | 0.123518164 |
|  |  | *Euphorbia helioscopia* | 0.0417 | 0.307 | 0.135830619 |
|  |  | *Manihot esculenta* | 0.0323 | 0.2766 | 0.116775127 |
|  |  | *Antidesma bunius* | 0.031 | 0.3425 | 0.090510949 |
|  |  | *Breynia fruticosa* | 0.0284 | 0.3307 | 0.08587844 |
|  |  | *Glochidion chodoense* | 0.0291 | 0.3267 | 0.089072544 |
|  |  | *Phyllanthus urinaria* | 0.0277 | 0.3402 | 0.081422693 |
| 45 | ***rpoB*** | *Ricinus communis* | 0.0324 | 0.2879 | 0.112539076 |
|  |  | *Jatropha curcas* | 0.0335 | 0.279 | 0.120071685 |
|  |  | *Euphorbia helioscopia* | 0.0382 | 0.318 | 0.120125786 |
|  |  | *Manihot esculenta* | 0.032 | 0.2798 | 0.114367405 |
|  |  | *Antidesma bunius* | 0.038 | 0.3125 | 0.1216 |
|  |  | *Breynia fruticosa* | 0.0347 | 0.3295 | 0.105311077 |
|  |  | *Glochidion chodoense* | 0.0367 | 0.3313 | 0.110775732 |
|  |  | *Phyllanthus urinaria* | 0.0376 | 0.3143 | 0.119630926 |
| 46 | ***psbM*** | *Ricinus communis* | 0.0131 | 0.4951 | 0.026459301 |
|  |  | *Jatropha curcas* | 0.0265 | 0.5659 | 0.046828061 |
|  |  | *Euphorbia helioscopia* | 0.0265 | 0.4135 | 0.064087062 |
|  |  | *Manihot esculenta* | 0.0265 | 0.4135 | 0.064087062 |
|  |  | *Antidesma bunius* | 0.0263 | 0.5047 | 0.052110164 |
|  |  | *Breynia fruticosa* | 0.0131 | 0.4951 | 0.026459301 |
|  |  | *Glochidion chodoense* | 0.0131 | 0.4951 | 0.026459301 |
|  |  | *Phyllanthus urinaria* | 0.0131 | 0.4951 | 0.026459301 |
| 47 | ***rps14*** | *Ricinus communis* | 0.0175 | 0.279 | 0.062724014 |
|  |  | *Jatropha curcas* | 0.0264 | 0.1981 | 0.133266027 |
|  |  | *Euphorbia helioscopia* | 0.0309 | 0.3005 | 0.102828619 |
|  |  | *Manihot esculenta* | 0.0219 | 0.2605 | 0.084069098 |
|  |  | *Antidesma bunius* | 0.0131 | 0.2194 | 0.059708295 |
|  |  | *Breynia fruticosa* | 0.0309 | 0.324 | 0.09537037 |
|  |  | *Glochidion chodoense* | 0.0263 | 0.3501 | 0.075121394 |
|  |  | *Phyllanthus urinaria* | 0.0219 | 0.324 | 0.067592593 |
| 48 | ***psaB*** | *Ricinus communis* | 0.0095 | 0.305 | 0.031147541 |
|  |  | *Jatropha curcas* | 0.0119 | 0.3228 | 0.036864932 |
|  |  | *Euphorbia helioscopia* | 0.0095 | 0.3377 | 0.028131478 |
|  |  | *Manihot esculenta* | 0.0101 | 0.2989 | 0.033790565 |
|  |  | *Antidesma bunius* | 0.0089 | 0.2983 | 0.029835736 |
|  |  | *Breynia fruticosa* | 0.0119 | 0.3602 | 0.033037202 |
|  |  | *Glochidion chodoense* | 0.014 | 0.343 | 0.040816327 |
|  |  | *Phyllanthus urinaria* | 0.0125 | 0.3472 | 0.036002304 |
| 49 | ***psaA*** | *Ricinus communis* | 0.0102 | 0.2739 | 0.037239869 |
|  |  | *Jatropha curcas* | 0.0088 | 0.2492 | 0.035313002 |
|  |  | *Euphorbia helioscopia* | 0.0088 | 0.2844 | 0.030942335 |
|  |  | *Manihot esculenta* | 0.0094 | 0.2541 | 0.03699331 |
|  |  | *Antidesma bunius* | 0.0094 | 0.2928 | 0.032103825 |
|  |  | *Breynia fruticosa* | 0.0088 | 0.3013 | 0.029206771 |
|  |  | *Glochidion chodoense* | 0.0082 | 0.3072 | 0.026692708 |
|  |  | *Phyllanthus urinaria* | 0.0093 | 0.296 | 0.031418919 |
| 50 | ***ndhJ*** | *Ricinus communis* | 0.0223 | 0.3569 | 0.062482488 |
|  |  | *Jatropha curcas* | 0.0208 | 0.3075 | 0.067642276 |
|  |  | *Euphorbia helioscopia* | 0.0408 | 0.3672 | 0.111111111 |
|  |  | *Manihot esculenta* | 0.0208 | 0.3093 | 0.067248626 |
|  |  | *Antidesma bunius* | 0.0237 | 0.365 | 0.064931507 |
|  |  | *Breynia fruticosa* | 0.0379 | 0.3661 | 0.103523627 |
|  |  | *Glochidion chodoense* | 0.0379 | 0.3512 | 0.107915718 |
|  |  | *Phyllanthus urinaria* | 0.0379 | 0.3661 | 0.103523627 |
| 51 | ***ndhC*** | *Ricinus communis* | 0.0222 | 0.2321 | 0.095648427 |
|  |  | *Jatropha curcas* | 0.0297 | 0.2494 | 0.119085806 |
|  |  | *Euphorbia helioscopia* | 0 | 0 | 0 |
|  |  | *Manihot esculenta* | 0.0259 | 0.1847 | 0.140227396 |
|  |  | *Antidesma bunius* | 0.0147 | 0.2477 | 0.059345983 |
|  |  | *Breynia fruticosa* | 0.0335 | 0.2321 | 0.144334339 |
|  |  | *Glochidion chodoense* | 0.0297 | 0.2017 | 0.147248389 |
|  |  | *Phyllanthus urinaria* | 0.0222 | 0.2007 | 0.110612855 |
| 52 | ***atpB*** | *Ricinus communis* | 0.0176 | 0.3726 | 0.047235641 |
|  |  | *Jatropha curcas* | 0.0173 | 0.3318 | 0.052139843 |
|  |  | *Euphorbia helioscopia* | 0.0213 | 0.3508 | 0.060718358 |
|  |  | *Manihot esculenta* | 0.014 | 0.3262 | 0.042918455 |
|  |  | *Antidesma bunius* | 0.0194 | 0.3833 | 0.050613097 |
|  |  | *Breynia fruticosa* | 0.0176 | 0.4089 | 0.043042309 |
|  |  | *Glochidion chodoense* | 0.0176 | 0.4136 | 0.042553191 |
|  |  | *Phyllanthus urinaria* | 0.0176 | 0.4092 | 0.043010753 |
| 53 | ***psaI*** | *Ricinus communis* | 0.1468 | 0.3418 | 0.42949093 |
|  |  | *Jatropha curcas* | 0.1184 | 0.3362 | 0.352171327 |
|  |  | *Euphorbia helioscopia* | 0.1468 | 0.2824 | 0.519830028 |
|  |  | *Manihot esculenta* | 0.1468 | 0.2824 | 0.519830028 |
|  |  | *Antidesma bunius* | 0.1277 | 0.3362 | 0.379833432 |
|  |  | *Breynia fruticosa* | 0.1557 | 0.2801 | 0.555872903 |
|  |  | *Glochidion chodoense* | 0.1557 | 0.2801 | 0.555872903 |
|  |  | *Phyllanthus urinaria* | 0.1557 | 0.2801 | 0.555872903 |
| 54 | ***petA*** | *Ricinus communis* | 0.0349 | 0.3619 | 0.096435479 |
|  |  | *Jatropha curcas* | 0.0321 | 0.4039 | 0.079475118 |
|  |  | *Euphorbia helioscopia* | 0.0398 | 0.4222 | 0.094268119 |
|  |  | *Manihot esculenta* | 0.0292 | 0.3206 | 0.091079226 |
|  |  | *Antidesma bunius* | 0.0329 | 0.3775 | 0.087152318 |
|  |  | *Breynia fruticosa* | 0.0359 | 0.4426 | 0.081111613 |
|  |  | *Glochidion chodoense* | 0.0403 | 0.4404 | 0.09150772 |
|  |  | *Phyllanthus urinaria* | 0.0395 | 0.4548 | 0.086851363 |
| 55 | ***psbJ*** | *Ricinus communis* | 0 | 0.2516 | 0 |
|  |  | *Jatropha curcas* | 0 | 0.2516 | 0 |
|  |  | *Euphorbia helioscopia* | 0.0234 | 0.3379 | 0.069251258 |
|  |  | *Manihot esculenta* | 0.0116 | 0.2088 | 0.055555556 |
|  |  | *Antidesma bunius* | 0.0232 | 0.1722 | 0.134727062 |
|  |  | *Breynia fruticosa* | 0.0115 | 0.1337 | 0.086013463 |
|  |  | *Glochidion chodoense* | 0.0115 | 0.1712 | 0.067172897 |
|  |  | *Phyllanthus urinaria* | 0.0115 | 0.1337 | 0.086013463 |
| 56 | ***psbL*** | *Ricinus communis* | 0.0113 | 0.1786 | 0.063269877 |
|  |  | *Jatropha curcas* | 0.0113 | 0.2308 | 0.048960139 |
|  |  | *Euphorbia helioscopia* | 0.0113 | 0.2308 | 0.048960139 |
|  |  | *Manihot esculenta* | 0.0113 | 0.2308 | 0.048960139 |
|  |  | *Antidesma bunius* | 0.0347 | 0.128 | 0.27109375 |
|  |  | *Breynia fruticosa* | 0.0113 | 0.1786 | 0.063269877 |
|  |  | *Glochidion chodoense* | 0.0113 | 0.1298 | 0.087057011 |
|  |  | *Phyllanthus urinaria* | 0.0113 | 0.1786 | 0.063269877 |
| 57 | ***psaJ*** | *Ricinus communis* | 0.0206 | 0.4319 | 0.047696226 |
|  |  | *Jatropha curcas* | 0.0102 | 0.3326 | 0.030667468 |
|  |  | *Euphorbia helioscopia* | 0.0418 | 0.4291 | 0.09741319 |
|  |  | *Manihot esculenta* | 0.0205 | 0.3368 | 0.060866983 |
|  |  | *Antidesma bunius* | 0.0206 | 0.3782 | 0.054468535 |
|  |  | *Breynia fruticosa* | 0 | 0.4939 | 0 |
|  |  | *Glochidion chodoense* | 0 | 0.5546 | 0 |
|  |  | *Phyllanthus urinaria* | 0 | 0.5546 | 0 |
| 58 | ***rpl33*** | *Ricinus communis* | 0.0551 | 0.3719 | 0.148158107 |
|  |  | *Jatropha curcas* | 0.0482 | 0.3648 | 0.132127193 |
|  |  | *Euphorbia helioscopia* | 0.0412 | 0.5638 | 0.073075559 |
|  |  | *Manihot esculenta* | 0.092 | 0.3657 | 0.251572327 |
|  |  | *Antidesma bunius* | 0.0885 | 0.3795 | 0.233201581 |
|  |  | *Breynia fruticosa* | 0.0659 | 0.3168 | 0.208017677 |
|  |  | *Glochidion chodoense* | 0.0588 | 0.3494 | 0.168288495 |
|  |  | *Phyllanthus urinaria* | 0.0661 | 0.3859 | 0.171287898 |
| 59 | ***rpl20*** | *Ricinus communis* | 0.0865 | 0.1427 | 0.606166783 |
|  |  | *Jatropha curcas* | 0.1161 | 0.1151 | 1.008688097 |
|  |  | *Euphorbia helioscopia* | 0.1078 | 0.1417 | 0.760762174 |
|  |  | *Manihot esculenta* | 0.0911 | 0.199 | 0.457788945 |
|  |  | *Antidesma bunius* | 0.099 | 0.1872 | 0.528846154 |
|  |  | *Breynia fruticosa* | 0.1153 | 0.2239 | 0.514962037 |
|  |  | *Glochidion chodoense* | 0.1068 | 0.2398 | 0.445371143 |
|  |  | *Phyllanthus urinaria* | 0.1069 | 0.2228 | 0.479802513 |
| 60 | ***rps12*** | *Ricinus communis* | 0.0307 | 0.075 | 0.409333333 |
|  |  | *Jatropha curcas* | 0.0272 | 0.0324 | 0.839506173 |
|  |  | *Euphorbia helioscopia* | 0.014 | 0.0515 | 0.27184466 |
|  |  | *Manihot esculenta* | 0.0084 | 0.024 | 0.35 |
|  |  | *Antidesma bunius* | 0.011 | 0.099 | 0.111111111 |
|  |  | *Breynia fruticosa* | 0.011 | 0.1352 | 0.081360947 |
|  |  | *Glochidion chodoense* | 0.0073 | 0.123 | 0.059349593 |
|  |  | *Phyllanthus urinaria* | 0.011 | 0.1109 | 0.099188458 |
| 61 | ***psbB*** | *Ricinus communis* | 0.0065 | 0.4413 | 0.014729209 |
|  |  | *Jatropha curcas* | 0.0048 | 0.3832 | 0.012526096 |
|  |  | *Euphorbia helioscopia* | 0.0095 | 0.4596 | 0.020670148 |
|  |  | *Manihot esculenta* | 0.0048 | 0.3789 | 0.01266825 |
|  |  | *Antidesma bunius* | 0.0091 | 0.4162 | 0.021864488 |
|  |  | *Breynia fruticosa* | 0.0109 | 0.4358 | 0.025011473 |
|  |  | *Glochidion chodoense* | 0.0109 | 0.4074 | 0.026755032 |
|  |  | *Phyllanthus urinaria* | 0.0117 | 0.4459 | 0.026239067 |
| 62 | ***rpoA*** | *Ricinus communis* | 0.0794 | 0.2955 | 0.268697124 |
|  |  | *Jatropha curcas* | 0.0791 | 0.295 | 0.268135593 |
|  |  | *Euphorbia helioscopia* | 0.0951 | 0.3175 | 0.299527559 |
|  |  | *Manihot esculenta* | 0.0772 | 0.3246 | 0.237831177 |
|  |  | *Antidesma bunius* | 0.083 | 0.3259 | 0.254679349 |
|  |  | *Breynia fruticosa* | 0.0772 | 0.303 | 0.254785479 |
|  |  | *Glochidion chodoense* | 0.0804 | 0.3038 | 0.264647795 |
|  |  | *Phyllanthus urinaria* | 0.0793 | 0.3446 | 0.23012188 |
| 63 | ***rps11*** | *Ricinus communis* | 0.0332 | 0.5081 | 0.065341468 |
|  |  | *Jatropha curcas* | 0.0433 | 0.6106 | 0.070913855 |
|  |  | *Euphorbia helioscopia* | 0.0419 | 0.5844 | 0.071697467 |
|  |  | *Manihot esculenta* | 0.0366 | 0.4692 | 0.078005115 |
|  |  | *Antidesma bunius* | 0.0555 | 0.4475 | 0.124022346 |
|  |  | *Breynia fruticosa* | 0.0574 | 0.4163 | 0.137881336 |
|  |  | *Glochidion chodoense* | 0.0538 | 0.4537 | 0.11858056 |
|  |  | *Phyllanthus urinaria* | 0.0503 | 0.4908 | 0.102485738 |
| 64 | ***rps8*** | *Ricinus communis* | 0.0568 | 0.4798 | 0.118382659 |
|  |  | *Jatropha curcas* | 0.0675 | 0.3919 | 0.172237816 |
|  |  | *Euphorbia helioscopia* | 0.0604 | 0.4781 | 0.126333403 |
|  |  | *Manihot esculenta* | 0.0461 | 0.4438 | 0.10387562 |
|  |  | *Antidesma bunius* | 0.0805 | 0.3468 | 0.232122261 |
|  |  | *Breynia fruticosa* | 0.0859 | 0.3748 | 0.229188901 |
|  |  | *Glochidion chodoense* | 0.0896 | 0.3585 | 0.249930265 |
|  |  | *Phyllanthus urinaria* | 0.0913 | 0.3856 | 0.236773859 |
| 65 | ***rps3*** | *Ricinus communis* | 0.0629 | 0.5429 | 0.115859274 |
|  |  | *Jatropha curcas* | 0.0914 | 0.5105 | 0.179040157 |
|  |  | *Euphorbia helioscopia* | 0.063 | 0.514 | 0.122568093 |
|  |  | *Manihot esculenta* | 0.0776 | 0.5107 | 0.151948306 |
|  |  | *Antidesma bunius* | 0.0845 | 0.5388 | 0.156829993 |
|  |  | *Breynia fruticosa* | 0.0858 | 0.5262 | 0.163055872 |
|  |  | *Glochidion chodoense* | 0.0856 | 0.4895 | 0.174872319 |
|  |  | *Phyllanthus urinaria* | 0.0853 | 0.5324 | 0.160217881 |
| 66 | ***rpl22*** | *Ricinus communis* | 0.2443 | 0.665 | 0.367368421 |
|  |  | *Jatropha curcas* | 0.1563 | 0.3958 | 0.394896412 |
|  |  | *Euphorbia helioscopia* | 0.1715 | 0.4189 | 0.409405586 |
|  |  | *Manihot esculenta* | 0.1652 | 0.3563 | 0.463654224 |
|  |  | *Antidesma bunius* | 0.2148 | 0.7975 | 0.269341693 |
|  |  | *Breynia fruticosa* | 0.2944 | 0.5738 | 0.513070756 |
|  |  | *Glochidion chodoense* | 0.2518 | 0.701 | 0.359201141 |
|  |  | *Phyllanthus urinaria* | 0.2498 | 0.7464 | 0.334673098 |
| 67 | ***rps19*** | *Ricinus communis* | 0.0191 | 0.4092 | 0.046676442 |
|  |  | *Jatropha curcas* | 0.0435 | 0.44 | 0.098863636 |
|  |  | *Euphorbia helioscopia* | 0.0239 | 0.4993 | 0.047867014 |
|  |  | *Manihot esculenta* | 0.0263 | 0.4832 | 0.054428808 |
|  |  | *Antidesma bunius* | 0.024 | 0.3779 | 0.063508865 |
|  |  | *Breynia fruticosa* | 0.0338 | 0.4315 | 0.078331402 |
|  |  | *Glochidion chodoense* | 0.0338 | 0.4285 | 0.078879813 |
|  |  | *Phyllanthus urinaria* | 0.0362 | 0.4222 | 0.085741355 |
| 68 | ***rpl2*** | *Ricinus communis* | 0.0214 | 0.0505 | 0.423762376 |
|  |  | *Jatropha curcas* | 0.0214 | 0.0661 | 0.323751891 |
|  |  | *Euphorbia helioscopia* | 0.0197 | 0.0662 | 0.297583082 |
|  |  | *Manihot esculenta* | 0.0214 | 0.0556 | 0.384892086 |
|  |  | *Antidesma bunius* | 0.0247 | 0.0453 | 0.545253863 |
|  |  | *Breynia fruticosa* | 0.0197 | 0.0557 | 0.353680431 |
|  |  | *Glochidion chodoense* | 0.0197 | 0.0557 | 0.353680431 |
|  |  | *Phyllanthus urinaria* | 0.0197 | 0.077 | 0.255844156 |
| 69 | ***ndhB*** | *Ricinus communis* | 0.0091 | 0.0486 | 0.187242798 |
|  |  | *Jatropha curcas* | 0.0118 | 0.0518 | 0.227799228 |
|  |  | *Euphorbia helioscopia* | 0.0091 | 0.0485 | 0.187628866 |
|  |  | *Manihot esculenta* | 0.01 | 0.0485 | 0.206185567 |
|  |  | *Antidesma bunius* | 0.0126 | 0.0458 | 0.27510917 |
|  |  | *Breynia fruticosa* | 0.01 | 0.0632 | 0.158227848 |
|  |  | *Glochidion chodoense* | 0.0109 | 0.0661 | 0.164901664 |
|  |  | *Phyllanthus urinaria* | 0.0109 | 0.0632 | 0.172468354 |
| 70 | ***ccsA*** | *Ricinus communis* | 0.1227 | 0.4054 | 0.302664036 |
|  |  | *Jatropha curcas* | 0.1134 | 0.4448 | 0.254946043 |
|  |  | *Euphorbia helioscopia* | 0.1357 | 0.4726 | 0.287134998 |
|  |  | *Manihot esculenta* | 0.1221 | 0.4276 | 0.28554724 |
|  |  | *Antidesma bunius* | 0.1285 | 0.4066 | 0.316035416 |
|  |  | *Breynia fruticosa* | 0.1305 | 0.4575 | 0.285245902 |
|  |  | *Glochidion chodoense* | 0.1327 | 0.4713 | 0.281561638 |
|  |  | *Phyllanthus urinaria* | 0.1297 | 0.4797 | 0.270377319 |
| 71 | ***ndhD*** | *Ricinus communis* | 0.0784 | 0.4421 | 0.177335444 |
|  |  | *Jatropha curcas* | 0.0833 | 0.4181 | 0.199234633 |
|  |  | *Euphorbia helioscopia* | 0.087 | 0.4656 | 0.18685567 |
|  |  | *Manihot esculenta* | 0.076 | 0.46 | 0.165217391 |
|  |  | *Antidesma bunius* | 0.0828 | 0.4459 | 0.185691859 |
|  |  | *Breynia fruticosa* | 0.0814 | 0.4904 | 0.165986949 |
|  |  | *Glochidion chodoense* | 0.0834 | 0.5048 | 0.165213946 |
|  |  | *Phyllanthus urinaria* | 0.0824 | 0.4872 | 0.169129721 |
| 72 | ***ndhG*** | *Ricinus communis* | 0.0742 | 0.2995 | 0.247746244 |
|  |  | *Jatropha curcas* | 0.0667 | 0.2633 | 0.253323205 |
|  |  | *Euphorbia helioscopia* | 0.0612 | 0.298 | 0.205369128 |
|  |  | *Manihot esculenta* | 0.0612 | 0.286 | 0.213986014 |
|  |  | *Antidesma bunius* | 0.057 | 0.2273 | 0.250769908 |
|  |  | *Breynia fruticosa* | 0.0584 | 0.2992 | 0.195187166 |
|  |  | *Glochidion chodoense* | 0.0583 | 0.2904 | 0.200757576 |
|  |  | *Phyllanthus urinaria* | 0.0747 | 0.2775 | 0.269189189 |
| 73 | ***ndhI*** | *Ricinus communis* | 0.0589 | 0.4767 | 0.123557793 |
|  |  | *Jatropha curcas* | 0.0628 | 0.4013 | 0.156491403 |
|  |  | *Euphorbia helioscopia* | 0.0429 | 0.4272 | 0.100421348 |
|  |  | *Manihot esculenta* | 0.0298 | 0.4153 | 0.071755358 |
|  |  | *Antidesma bunius* | 0.0306 | 0.3838 | 0.079729026 |
|  |  | *Breynia fruticosa* | 0.0372 | 0.4505 | 0.082574917 |
|  |  | *Glochidion chodoense* | 0.027 | 0.4848 | 0.055693069 |
|  |  | *Phyllanthus urinaria* | 0.0427 | 0.4784 | 0.089255853 |
| 74 | ***rps15*** | *Ricinus communis* | 0.0922 | 0.6055 | 0.152270851 |
|  |  | *Jatropha curcas* | 0.1167 | 0.6742 | 0.173094037 |
|  |  | *Euphorbia helioscopia* | 0.1163 | 0.7745 | 0.150161394 |
|  |  | *Manihot esculenta* | 0.0977 | 0.6422 | 0.152133292 |
|  |  | *Antidesma bunius* | 0.1135 | 0.6675 | 0.170037453 |
|  |  | *Breynia fruticosa* | 0.1027 | 0.7864 | 0.130595117 |
|  |  | *Glochidion chodoense* | 0.1135 | 0.7959 | 0.142605855 |
|  |  | *Phyllanthus urinaria* | 0.108 | 0.8426 | 0.128174697 |
| 75 | ***ycf1*** | *Ricinus communis* | 0.2737 | 0.3243 | 0.843971631 |
|  |  | *Jatropha curcas* | 0.2072 | 0.4104 | 0.504873294 |
|  |  | *Euphorbia helioscopia* | 0.2398 | 0.4528 | 0.52959364 |
|  |  | *Manihot esculenta* | 0.2653 | 0.4649 | 0.570660357 |
|  |  | *Antidesma bunius* | 0.2328 | 0.4023 | 0.578672632 |
|  |  | *Breynia fruticosa* | 0.2484 | 0.4978 | 0.498995581 |
|  |  | *Glochidion chodoense* | 0.2493 | 0.4827 | 0.516469857 |
|  |  | *Phyllanthus urinaria* | 0.2404 | 0.4762 | 0.504829903 |
| 76 | ***ycf2*** | *Ricinus communis* | 0.0357 | 0.0792 | 0.450757576 |
|  |  | *Jatropha curcas* | 0.0342 | 0.0782 | 0.437340153 |
|  |  | *Euphorbia helioscopia* | 0.0371 | 0.0803 | 0.462017435 |
|  |  | *Manihot esculenta* | 0.0371 | 0.0772 | 0.480569948 |
|  |  | *Antidesma bunius* | 0.0396 | 0.0833 | 0.475390156 |
|  |  | *Breynia fruticosa* | 0.0391 | 0.0897 | 0.435897436 |
|  |  | *Glochidion chodoense* | 0.0407 | 0.0944 | 0.431144068 |
|  |  | *Phyllanthus urinaria* | 0.0393 | 0.0902 | 0.435698448 |
| 77 | ***psbZ*** | *Ricinus communis* | 0.0292 | 0.2867 | 0.101848622 |
|  |  | *Jatropha curcas* | 0.0293 | 0.1946 | 0.150565262 |
|  |  | *Euphorbia helioscopia* | 0 | 0 | 0 |
|  |  | *Manihot esculenta* | 0.0219 | 0.1938 | 0.113003096 |
|  |  | *Antidesma bunius* | 0.0293 | 0.1405 | 0.208540925 |
|  |  | *Breynia fruticosa* | 0.0219 | 0.222 | 0.098648649 |
|  |  | *Glochidion chodoense* | 0 | 0 | 0 |
|  |  | *Phyllanthus urinaria* | 0.0219 | 0.2817 | 0.077742279 |
| 78 | ***petB*** | *Ricinus communis* | 0.0055 | 0.276 | 0.019927536 |
|  |  | *Jatropha curcas* | 0.0069 | 0.3317 | 0.020801929 |
|  |  | *Euphorbia helioscopia* | 0.0083 | 0.3505 | 0.023680456 |
|  |  | *Manihot esculenta* | 0.0055 | 0.3261 | 0.016865992 |
|  |  | *Antidesma bunius* | 0.0055 | 0.4347 | 0.012652404 |
|  |  | *Breynia fruticosa* | 0.0027 | 0.4355 | 0.00619977 |
|  |  | *Glochidion chodoense* | 0.0027 | 0.4355 | 0.00619977 |
|  |  | *Phyllanthus urinaria* | 0.0027 | 0.3925 | 0.006878981 |

**Supplementary table 10:** Nucleotide Diversity and Alignment Length in coding, non-coding, and intronic regions among *C. sabulosa* and four other Euphobiaceae species (*Ricinus communis*, *Jatropha curcas*, *Euphorbia helioscopia*, and *Manihot esculenta*).

| **Sr.No** | **Region** | **Location** | **Nucleotide Diversity** | **T. No's of Mutations** | **Region Length (all silent positions) excluding missing data** | **Alignment Length** |
| --- | --- | --- | --- | --- | --- | --- |
| 1 | *atpA* | CDS | 0.060630 | 221 | 1524 | 1524 |
| 2 | *atpB* | CDS | 0.056120 | 198 | 1488 | 1503 |
| 3 | *atpE* | CDS | 0.055970 | 54 | 402 | 402 |
| 4 | *atpF* | CDS | 0.058870 | 70 | 513 | 615 |
| 5 | *atpH* | CDS | 0.047150 | 27 | 246 | 246 |
| 6 | *atpI* | CDS | 0.056770 | 94 | 687 | 765 |
| 7 | *ccsA* | CDS | 0.104550 | 245 | 966 | 993 |
| 8 | *cemA* | CDS | 0.080490 | 134 | 687 | 699 |
| 9 | *clpP* | CDS | 0.062590 | 88 | 588 | 591 |
| 10 | *matK* | CDS | 0.116720 | 423 | 1495 | 1540 |
| 11 | *ndhA* | CDS | 0.073260 | 191 | 1092 | 1092 |
| 12 | *ndhB* | CDS | 0.011390 | 43 | 1527 | 1533 |
| 13 | *ndhD* | CDS | 0.092550 | 329 | 1503 | 1647 |
| 14 | *ndhE* | CDS | 0.059150 | 41 | 306 | 306 |
| 15 | *ndhF* | CDS | 0.109500 | 584 | 2222 | 2403 |
| 16 | *ndhG* | CDS | 0.071750 | 89 | 531 | 534 |
| 17 | *ndhH* | CDS | 0.068710 | 192 | 1176 | 1182 |
| 18 | *ndhI* | CDS | 0.066670 | 78 | 498 | 523 |
| 19 | *ndhJ* | CDS | 0.054300 | 62 | 477 | 477 |
| 20 | *petA* | CDS | 0.065210 | 151 | 963 | 963 |
| 21 | *petB* | CDS | 0.044790 | 51 | 489 | 687 |
| 22 | *petD* | CDS | 0.053000 | 56 | 483 | 601 |
| 23 | *psaA* | CDS | 0.041150 | 219 | 2253 | 2253 |
| 24 | *psaB* | CDS | 0.042090 | 223 | 2205 | 2205 |
| 25 | *psaC* | CDS | 0.054470 | 32 | 246 | 246 |
| 26 | *psbA* | CDS | 0.027210 | 68 | 1062 | 1062 |
| 27 | *psbB* | CDS | 0.054090 | 200 | 1527 | 1527 |
| 28 | *psbC* | CDS | 0.042420 | 142 | 1386 | 1422 |
| 29 | *psbD* | CDS | 0.034930 | 90 | 1062 | 1062 |
| 30 | *psbE* | CDS | 0.032540 | 20 | 252 | 252 |
| 31 | *psbH* | CDS | 0.063510 | 33 | 222 | 222 |
| 32 | *rbcL* | CDS | 0.057350 | 192 | 1428 | 1446 |
| 33 | *rpl2* | CDS | 0.019270 | 40 | 825 | 873 |
| 34 | *rpl14* | CDS | 0.065030 | 55 | 366 | 369 |
| 35 | *rpl16* | CDS | 0.078330 | 68 | 360 | 450 |
| 36 | *rpl20* | CDS | 0.078530 | 68 | 354 | 387 |
| 37 | *rpl22* | CDS | 0.117330 | 98 | 352 | 494 |
| 38 | *rpl23* | CDS | 0.014180 | 10 | 282 | 282 |
| 39 | *rpl33* | CDS | 0.087060 | 42 | 201 | 207 |
| 40 | *rpoA* | CDS | 0.081070 | 196 | 993 | 1032 |
| 41 | *rpoB* | CDS | 0.054250 | 419 | 3213 | 3219 |
| 42 | *rpoC1* | CDS | 0.05288 | 252 | 1965 | 2043 |
| 43 | *rpoC2* | CDS | 0.077540 | 766 | 4092 | 4263 |
| 44 | *rps2* | CDS | 0.057810 | 100 | 711 | 711 |
| 45 | *rps3* | CDS | 0.093760 | 147 | 657 | 666 |
| 46 | *rps4* | CDS | 0.055610 | 84 | 606 | 606 |
| 47 | *rps7* | CDS | 0.007690 | 9 | 468 | 468 |
| 48 | *rps8* | CDS | 0.081730 | 82 | 405 | 405 |
| 49 | *rps11* | CDS | 0.089210 | 87 | 417 | 417 |
| 50 | *rps12* | CDS | 0.025710 | 19 | 354 | 372 |
| 51 | *rps14* | CDS | 0.049830 | 34 | 303 | 303 |
| 52 | *rps15* | CDS | 0.109160 | 71 | 273 | 273 |
| 53 | *rps18* | CDS | 0.047890 | 33 | 284 | 328 |
| 54 | *rps19* | CDS | 0.060220 | 40 | 279 | 279 |
| 55 | *accD* | CDS | 0.084560 | 295 | 1457 | 1746 |
| 56 | *ycf1* | CDS | 0.141650 | 1647 | 4814 | 6204 |
| 57 | *ycf2* | CDS | 0.025160 | 417 | 6706 | 7059 |
| 58 | *ycf3* | CDS | 0.036710 | 46 | 504 | 510 |
| 59 | *ycf4* | CDS | 0.070810 | 94 | 555 | 636 |
| 60 | *psbK-psbI* | IGS | 0.178300 | 149 | 341 | 524 |
| 61 | *atpF-atpH* | IGS | 0.181900 | 151 | 337 | 753 |
| 62 | *atpH-atpI* | IGS | 0.177190 | 219 | 513 | 1428 |
| 63 | *rps2-rpoC2* | IGS | 0.147690 | 78 | 216 | 318 |
| 64 | *rpoC1 Intron* | Intron | 0.108490 | 183 | 695 | 938 |
| 65 | *petN-psbM* | IGS | 0.161310 | 312 | 791 | 1611 |
| 66 | *psaA-ycf3* | IGS | 0.166720 | 275 | 667 | 1390 |
| 67 | *ycf3 Intron 2* | Intron | 0.099360 | 154 | 628 | 816 |
| 68 | *ycf3 Intron 1* | Intron | 0.098950 | 162 | 667 | 826 |
| 69 | *atpB-rbcL* | IGS | 0.120550 | 206 | 691 | 915 |
| 70 | *rbcL-accD* | IGS | 0.136530 | 177 | 531 | 961 |
| 71 | *accD-psaI* | IGS | 0.197520 | 212 | 443 | 1685 |
| 72 | *psaI-ycf4* | IGS | 0.208920 | 78 | 157 | 548 |
| 73 | *ycf4-cemA* | IGS | 0.185460 | 205 | 447 | 1128 |
| 74 | *cemA-petA* | IGS | 0.137100 | 74 | 221 | 354 |
| 75 | *petA-psbJ* | IGS | 0.193300 | 280 | 597 | 1267 |
| 76 | *psbE-petL* | IGS | 0.188620 | 464 | 1019 | 2137 |
| 77 | *psaJ-rpl33* | IGS | 0.183840 | 158 | 359 | 587 |
| 78 | *rpl33-rps18* | IGS | 0.254940 | 139 | 233 | 560 |
| 79 | *rps18-rpl20* | IGS | 0.217500 | 106 | 200 | 407 |
| 80 | *rpl20-rps12* | IGS | 0.115370 | 193 | 709 | 924 |
| 81 | *clpP Intron 2* | Intron | 0.119180 | 153 | 537 | 790 |
| 82 | *clpP Intron 1* | Intron | 0.144070 | 263 | 767 | 1004 |
| 83 | *clpP-psbB* | IGS | 0.142660 | 125 | 361 | 610 |
| 84 | *psbH-petB* | IGS | 0.078690 | 12 | 61 | 1212 |
| 85 | *petB-petD* | IGS | 0.109340 | 49 | 182 | 1093 |
| 86 | *rpl16-rps3* | IGS | 0.203450 | 56 | 116 | 1542 |
| 87 | *ndhB Intron* | Intron | 0.012280 | 20 | 668 | 682 |
| 88 | *ndhB-rps7* | IGS | 0.038080 | 27 | 281 | 341 |
| 89 | *psaC-ndhE* | IGS | 0.187500 | 115 | 248 | 442 |
| 90 | *ndhE-ndhG* | IGS | 0.166850 | 74 | 181 | 283 |
| 91 | *ndhG-ndhI* | IGS | 0.213890 | 168 | 324 | 643 |
| 92 | *ndhA Intron* | Intron | 0.134570 | 309 | 946 | 1310 |
| 93 | *rps15-ycf1* | IGS | 0.213920 | 81 | 158 | 574 |

**Supplementary table 11:** Nucleotide Diversity and Alignment Length in coding, non-coding, and intronic regions among *C. sabulosa* and four Phyllanthaceae species (*Antidesma bunius*, *Breynia fruticosa*, *Glochidion chodoense, and Phyllanthus urinaria*).

| **Sr.No** | **Region** | **Location** | **Nucleotide Diversity** | **T. No's of Mutations** | **Region Length (all silent positions) excluding missing data** | **Alignment Length** |
| --- | --- | --- | --- | --- | --- | --- |
| 1 | *atpA* | CDS | 0.056760 | 196 | 1524 | 1524 |
| 2 | *atpB* | CDS | 0.055380 | 188 | 1497 | 1497 |
| 3 | *atpE* | CDS | 0.054980 | 51 | 402 | 402 |
| 4 | *atpF* | CDS | 0.062880 | 80 | 555 | 555 |
| 5 | *atpH* | CDS | 0.058940 | 31 | 246 | 246 |
| 6 | *atpI* | CDS | 0.055380 | 95 | 744 | 759 |
| 7 | *ccsA* | CDS | 0.110030 | 240 | 957 | 1149 |
| 8 | *cemA* | CDS | 0.080150 | 123 | 675 | 747 |
| 9 | *clpP* | CDS | 0.070240 | 97 | 588 | 591 |
| 10 | *matK* | CDS | 0.126980 | 446 | 1505 | 1609 |
| 11 | *ndhA* | CDS | 0.079120 | 199 | 1092 | 1110 |
| 12 | *ndhB* | CDS | 0.012590 | 44 | 1533 | 1533 |
| 13 | *ndhD* | CDS | 0.089410 | 312 | 1521 | 1647 |
| 14 | *ndhE* | CDS | 0.064030 | 44 | 303 | 306 |
| 15 | *ndhF* | CDS | 0.109200 | 560 | 2217 | 2280 |
| 16 | *ndhG* | CDS | 0.067980 | 83 | 531 | 564 |
| 17 | *ndhH* | CDS | 0.061840 | 165 | 1182 | 1182 |
| 18 | *ndhI* | CDS | 0.066460 | 74 | 492 | 513 |
| 19 | *ndhJ* | CDS | 0.060170 | 63 | 477 | 477 |
| 20 | *petA* | CDS | 0.066670 | 145 | 963 | 969 |
| 21 | *petB* | CDS | 0.050310 | 57 | 489 | 648 |
| 22 | *petD* | CDS | 0.049690 | 53 | 483 | 601 |
| 23 | *psaA* | CDS | 0.040750 | 208 | 2253 | 2253 |
| 24 | *psaB* | CDS | 0.045710 | 224 | 2205 | 2205 |
| 25 | *psaC* | CDS | 0.047150 | 25 | 246 | 246 |
| 26 | *psbA* | CDS | 0.029660 | 71 | 1062 | 1062 |
| 27 | *psbB* | CDS | 0.053900 | 186 | 1527 | 1527 |
| 28 | *psbC* | CDS | 0.041770 | 133 | 1422 | 1422 |
| 29 | *psbD* | CDS | 0.036250 | 86 | 1062 | 1062 |
| 30 | *psbE* | CDS | 0.029760 | 18 | 252 | 252 |
| 31 | *psbH* | CDS | 0.047300 | 24 | 222 | 273 |
| 32 | *rbcL* | CDS | 0.059940 | 197 | 1428 | 1467 |
| 33 | *rpl2* | CDS | 0.017580 | 34 | 825 | 873 |
| 34 | *rpl14* | CDS | 0.068030 | 56 | 366 | 369 |
| 35 | *rpl16* | CDS | 0.085050 | 77 | 408 | 453 |
| 36 | *rpl20* | CDS | 0.081920 | 66 | 354 | 387 |
| 37 | *rpl22* | CDS | 0.207220 | 90 | 194 | 481 |
| 38 | *rpl23* | CDS | 0.026240 | 15 | 282 | 282 |
| 39 | *rpl33* | CDS | 0.076120 | 38 | 201 | 207 |
| 40 | *rpoA* | CDS | 0.072140 | 168 | 1005 | 1053 |
| 41 | *rpoB* | CDS | 0.059320 | 430 | 3213 | 3213 |
| 42 | *rpoC1* | CDS | 0.056690 | 252 | 1958 | 2076 |
| 43 | *rpoC2* | CDS | 0.080240 | 761 | 4074 | 4323 |
| 44 | *rps2* | CDS | 0.066100 | 110 | 711 | 711 |
| 45 | *rps3* | CDS | 0.093460 | 143 | 657 | 657 |
| 46 | *rps4* | CDS | 0.059570 | 82 | 606 | 606 |
| 47 | *rps7* | CDS | 0.006840 | 8 | 468 | 468 |
| 48 | *rps8* | CDS | 0.087900 | 81 | 405 | 405 |
| 49 | *rps11* | CDS | 0.078420 | 76 | 417 | 417 |
| 50 | *rps12* | CDS | 0.026880 | 20 | 372 | 372 |
| 51 | *rps14* | CDS | 0.051820 | 35 | 303 | 303 |
| 52 | *rps15* | CDS | 0.105130 | 67 | 273 | 273 |
| 53 | *rps18* | CDS | 0.059480 | 41 | 306 | 312 |
| 54 | *rps19* | CDS | 0.058420 | 36 | 279 | 279 |
| 55 | *accD* | CDS | 0.096370 | 325 | 1461 | 1530 |
| 56 | *ycf1* | CDS | 0.151460 | 1880 | 5350 | 5919 |
| 57 | *ycf2* | CDS | 0.029350 | 453 | 6759 | 6987 |
| 58 | *ycf3* | CDS | 0.036090 | 43 | 507 | 579 |
| 59 | *ycf4* | CDS | 0.073220 | 90 | 549 | 636 |
| 60 | *psbK-psbI* | IGS | 0.162640 | 96 | 265 | 541 |
| 61 | *atpF-atpH* | IGS | 0.180900 | 109 | 267 | 539 |
| 62 | *atpH-atpI* | IGS | 0.164910 | 256 | 664 | 1309 |
| 63 | *rps2-rpoC2* | IGS | 0.183890 | 87 | 211 | 292 |
| 64 | *rpoC1 Intron* | Intron | 0.110570 | 176 | 681 | 940 |
| 65 | *petN-psbM* | IGS | 0.161700 | 142 | 376 | 1307 |
| 66 | *psaA-ycf3* | IGS | 0.132460 | 190 | 610 | 1230 |
| 67 | *ycf3 Intron 2* | Intron | 0.100000 | 150 | 634 | 829 |
| 68 | *ycf3 Intron 1* | Intron | 0.098950 | 154 | 668 | 810 |
| 69 | *atpB-rbcL* | IGS | 0.132160 | 214 | 684 | 933 |
| 70 | *rbcL-accD* | IGS | 0.145170 | 180 | 538 | 976 |
| 71 | *accD-psaI* | IGS | 0.180420 | 186 | 429 | 823 |
| 72 | *psaI-ycf4* | IGS | 0.222220 | 126 | 234 | 421 |
| 73 | *ycf4-cemA* | IGS | 0.219440 | 354 | 674 | 1108 |
| 74 | *cemA-petA* | IGS | 0.134160 | 63 | 202 | 270 |
| 75 | *petA-psbJ* | IGS | 0.192240 | 219 | 477 | 1292 |
| 76 | *psbE-petL* | IGS | 0.143860 | 159 | 472 | 1591 |
| 77 | *psaJ-rpl33* | IGS | 0.182090 | 161 | 374 | 570 |
| 78 | *rpl33-rps18* | IGS | 0.208370 | 96 | 203 | 466 |
| 79 | *rps18-rpl20* | IGS | 0.208260 | 111 | 230 | 339 |
| 80 | *rpl20-rps12* | IGS | 0.115460 | 191 | 718 | 876 |
| 81 | *clpP Intron 2* | Intron | 0.110260 | 127 | 497 | 671 |
| 82 | *clpP Intron 1* | Intron | 0.133030 | 239 | 757 | 948 |
| 83 | *clpP-psbB* | IGS | 0.137080 | 126 | 383 | 604 |
| 84 | *psbH-petB* | IGS | 0.155000 | 45 | 120 | 1146 |
| 85 | *petB-petD* | IGS | 0.125000 | 56 | 188 | 1042 |
| 86 | *rpl16-rps3* | IGS | 0.165480 | 65 | 168 | 1134 |
| 87 | *ndhB Intron* | Intron | 0.021950 | 33 | 665 | 682 |
| 88 | *ndhB-rps7* | IGS | 0.040610 | 29 | 293 | 345 |
| 89 | *psaC-ndhE* | IGS | 0.170590 | 93 | 238 | 444 |
| 90 | *ndhE-ndhG* | IGS | 0.185880 | 78 | 177 | 259 |
| 91 | *ndhG-ndhI* | IGS | 0.240480 | 48 | 84 | 489 |
| 92 | *ndhA Intron* | Intron | 0.162550 | 367 | 988 | 1294 |
| 93 | *rps15-ycf1* | IGS | 0.246720 | 161 | 274 | 546 |

**Supplementary table 12:** The *C. Sabulosa* and 30 selected NCBI genomes of Euphorbiaceae and Phyllanthaceae for Phylogenetic tree analysis.

| **Family** | **Sub family** | **Tribe Name** | **Species** | **Gene bank accession number** |
| --- | --- | --- | --- | --- |
| **Euphorbiaceae** | **Acalyphoideae** | **Chrozophoreae** | *Chrozophora sabulosa* | MW541931 |
|  |  | **Epiprineae** | *Cleidiocarpon cavaleriei* | MG813873 |
|  |  | **Acalypheae** | *Ricinus communis* | JF937588 |
|  |  |  | *Cleidion brevipetiolatum* | OL804290 |
|  |  |  | *Mallotus japonicus* | MW244068 |
|  |  |  | *Macaranga tanarius* | MW297079 |
|  |  | **Plukenetieae** | *Plukenetia volubilis* | MW591569 |
|  | **Crotonoideae** | **Crotoneae** | *Croton laevigatus* | MN713923 |
|  |  | **Micrandreae** | *Hevea camargoana* | MN781109 |
|  |  | **Jatropheae** | *Jatropha curcas* | FJ695500 |
|  |  |  | *Deutzianthus tonkinensis* | MH933861 |
|  |  | **Manihoteae** | *Cnidoscolus aconitifolius* | MZ045411 |
|  |  |  | *Manihot esculenta* | NC010433 |
|  |  | **Aleuritideae** | *Vernicia montana* | MW297080 |
|  |  |  | *Aleurites moluccanus* | MW322810 |
|  | **Euphorbioideae** | **Euphorbieae** | *Euphorbia helioscopia* | MN199031 |
|  |  | **Hippomaneae** | *Balakata baccata* | MW266130 |
|  |  |  | *Triadica sebifera* | MT424756 |
|  |  |  | *Excoecaria agallocha* | MZ687828 |
| **Phyllanthaceae** | **Antidesmatoideae** | **Antidesmateae** | *Antidesma bunius* | ON022043 |
|  |  | **Scepeae** | *Baccaurea ramiflora* | MT900598 |
|  |  | **Bischofieae** | *Bischofia polycarpa* | MZ826267 |
|  | **Phyllanthoideae** | **Bridelieae** | *Bridelia tomentosa* | MW357611 |
|  |  | **Poranthereae** | *Leptopus cordifolius* | MZ424188 |
|  |  |  | *Flueggea virosa* | MT424755 |
|  |  |  | *Phyllanthus emblica* | OL693862 |
|  |  |  | *Sauropus spatulifolius* | MT089915 |
|  |  |  | *Breynia fruticosa* | MT863745 |
|  |  |  | *Glochidion chodoense* | MK056235 |
| **Anacardiaceae (OUT GROUP)** | | | *Mangifera indica* | KX871231 |
|  |  |  | *Lannea coromandelica* | MZ929419 |
